# The Amino-Terminal Region of Hepatitis E Virus ORF1 Containing a Methyltransferase (Met) and a Papain-Like Cysteine Protease (PCP) Domain Counteracts Type I Interferon Response

**DOI:** 10.3390/v10120726

**Published:** 2018-12-18

**Authors:** Eugénie Bagdassarian, Virginie Doceul, Marie Pellerin, Antonin Demange, Léa Meyer, Nolwenn Jouvenet, Nicole Pavio

**Affiliations:** 1 Anses, UMR 1161 Virologie, Laboratoire de Santé Animale, 94700 Maisons-Alfort, France; eugenie.bagdassarian@gmail.com (E.B.); marie.pellerin@anses.fr (M.P.); antonin.demange@ird.fr (A.D.); lea.meyer@vet-alfort.fr (L.M.); nicole.pavio@anses.fr (N.P.); 2 INRA, UMR 1161 Virologie, 94700 Maisons-Alfort, France; 3 École Nationale Vétérinaire d’Alfort, UMR 1161 Virologie, 94700 Maisons-Alfort, France; 4 CNRS-UMR3569, Unité de Génomique Virale et Vaccination, Institut Pasteur, 75015 Paris, France; nolwenn.jouvenet@pasteur.fr

**Keywords:** hepatitis E virus, innate immunity, interferon response, JAK/STAT pathway, zoonosis, emerging pathogen

## Abstract

Hepatitis E virus (HEV) is responsible for large waterborne epidemics of hepatitis in endemic countries and is an emerging zoonotic pathogen worldwide. In endemic regions, HEV-1 or HEV-2 genotypes are frequently associated with fulminant hepatitis in pregnant women, while with zoonotic HEV (HEV-3 and HEV-4), chronic cases of hepatitis and severe neurological disorders are reported. Hence, it is important to characterize the interactions between HEV and its host. Here, we investigated the ability of the nonstructural polyprotein encoded by the first open reading frame (*ORF1*) of HEV to modulate the host early antiviral response and, in particular, the type I interferon (IFN-I) system. We found that the amino-terminal region of HEV-3 ORF1 (MetYPCP), containing a putative methyltransferase (Met) and a papain-like cysteine protease (PCP) functional domain, inhibited IFN-stimulated response element (ISRE) promoter activation and the expression of several IFN-stimulated genes (ISGs) in response to IFN-I. We showed that the MetYPCP domain interfered with the Janus kinase (JAK)/signal transducer and activator of the transcription protein (STAT) signalling pathway by inhibiting STAT1 nuclear translocation and phosphorylation after IFN-I treatment. In contrast, MetYPCP had no effect on STAT2 phosphorylation and a limited impact on the activation of the JAK/STAT pathway after IFN-II stimulation. This inhibitory function seemed to be genotype-dependent, as MetYPCP from HEV-1 had no significant effect on the JAK/STAT pathway. Overall, this study provides evidence that the predicted MetYPCP domain of HEV ORF1 antagonises STAT1 activation to modulate the IFN response.

## 1. Introduction

Hepatitis E virus (HEV) is a single-stranded positive RNA virus belonging to the *Orthohepevirus* genus within the Hepeviridae family [1]. Its genome is 7.2 kb in length and codes for three open reading frames (*ORF1* to *3*) [2]. *ORF1* codes for a nonstructural polyprotein composed of several putative functional domains including a methyltransferase (Met), a domain of unknown function (Y), a papain-like cysteine protease (PCP), a macrodomain (X), a helicase and an RNA-dependent RNA polymerase (RdRp) [3]. It is still unclear whether ORF1 is expressed as a single polyprotein or cleaved to several functional proteins in the context of infection. Multiple studies have suggested that ORF1 is cleaved into several products [4,5,6,7,8], whereas a few others have reported a lack of processing of the viral polyprotein [9,10,11]. The use of different expression systems may explain these conflicting results. Recently, a paper has suggested that ORF1 is cleaved by thrombin and factor Xa [12]. *ORF2* and *ORF3* code for the capsid protein and a multifunctional phosphoprotein, respectively. Four genotypes infect humans. Genotypes 1 and 2 (HEV-1 and HEV-2) are transmitted via the faecal-oral route, through the consumption of contaminated water or soiled food in endemic regions. In contrast, genotypes 3 and 4 (HEV-3 and HEV-4) are detected in humans and other animal species worldwide and are transmitted via direct contact with infected animals or the consumption of infected meat [13,14]. In most human cases, HEV infection causes an acute hepatitis that is self-limited. However, fulminant hepatic failure can occur in pregnant women in endemic regions (HEV-1 or -2), in patients with underlying chronic liver disease, or in the elderly (HEV-3 or -4). More recently, chronic cases of hepatitis E have been reported in immunocompromised patients (HEV-3 or HEV-4) and extrahepatic manifestations including renal, pancreatic and neurological disorders have been linked to HEV infection [15]. With the exception of China, no country has yet commercialized an HEV vaccine, and no treatment against HEV infection is approved.

Interferons (IFNs) are a group of secreted cytokines that play a key role in the host early antiviral response. Type I IFNs (IFN-I), composed mainly of IFN-α and β, are produced directly in response to viral infection, upon the sensing of viral molecular signatures by specialized cellular receptors such as retinoic-acid-inducible gene (RIG)-I-like receptors (RLRs) and Toll-like receptors (TLRs). IFN-I subsequently binds to IFN-α/β receptors (IFNAR) at the cell surface and activates the Janus kinase (JAK)/signal transducer and activator of transcription protein (STAT) signalling pathway in an autocrine and paracrine manner. The binding of IFN-I to receptors leads to the phosphorylation of tyrosine kinase 2 (TYK2) and JAK1 [16,17,18] and the subsequent phosphorylation of the cytoplasmic domain of the IFNAR subunits [18,19,20,21,22]. STAT1 and STAT2 are then recruited and phosphorylated by the JAK kinases on tyrosine 701 and tyrosine 690, respectively [18,23]. Phosphorylated STAT1/STAT2 heterodimers are released in the cytoplasm, where they interact with IFN response factor 9 (IRF9) to form IFN-stimulated gene (ISG) factor 3 (ISGF3). This transcription factor translocates to the nucleus, where it binds to specific promoter elements called IFN-stimulated response elements (ISRE), leading to the upregulation of hundreds of IFN-stimulated genes (ISGs) that may display antiviral properties and contribute to the establishment of a rapid and robust antiviral state within the cell [24]. Most cells can produce IFN-I. In contrast, type II IFN (IFN-γ) is secreted mainly by activated T cells and natural killer cells. The binding of the cytokine to a specific IFN-γ receptor (IFNGR) leads to the phosphorylation of JAK1 and JAK2 and the subsequent phosphorylation of STAT1. STAT1 homodimers are then formed and translocate to the nucleus where they bind to specific promoters to activate the transcription of a different subset of ISGs [25].

Different reports have suggested that an IFN response is triggered by HEV as the expression of IFN-I, and multiple ISGs have been detected after infection in vivo and in vitro [26,27,28,29,30,31]. However, IFN-I seem to have a moderate and delayed antiviral effect on HEV infection in vitro and in patients in comparison, for instance, to the potent effect they exert on the hepatitis C virus (HCV), another hepatotropic RNA virus [32,33]. Consistently, recent studies have indicated that the host ISG response to IFN-I is inhibited during HEV infection [31,32,33,34], but the mechanisms involved in this inhibition remain poorly characterized. As a nonstructural polyprotein, HEV ORF1 contains one or several functional domains able to modulate the IFN-I system. The macrodomain, the PCP domain and the Met domain have been described as antagonists of the signalling cascade leading to IFN synthesis [35,36]. However, nothing is known about the ability of the viral polyprotein to inhibit the response to IFN-I and the JAK/STAT pathway. To address this question, we studied the effect of HEV ORF1 and several of its domains on this signalling pathway. We used a transfected cell model to express full-length or fragments of ORF1 fused to a FLAG tag, as it is difficult to detect the polyprotein and its putative cleavage products in the context of infection or replication [10,37]. We were particularly interested in testing PCP and the macrodomain (X), as such functional domains encoded by several RNA-positive viruses have been shown to modulate the host innate immune response [38,39,40,41,42,43]. The amino-terminal end of ORF1 (MetYPCP) containing Met, Y and PCP was also included in this study as a putative zinc finger domain is present in Met that might be critical for the enzymatic activity of PCP [44]. We found that the MetYPCP domain inhibited ISRE promoter activation and the expression of several ISGs after stimulation with IFN-I. Further investigations revealed that MetYPCP interfered with STAT1 nuclear translocation and phosphorylation. Overall, our data provides evidence that the predicted MetYPCP domain of HEV ORF1 antagonises STAT1 activation to modulate the IFN response.

## 2. Materials and Methods

### 2.1. Cells

Human embryonic kidney 293T cells were grown in Dulbecco’s modified Eagle’s medium (DMEM) supplemented with 10% heat-inactivated fetal calf serum (FCS), 1% pyruvate and 100 IU/mL penicillin and 100 µg/mL streptomycin (PS). Cells were maintained at 37 °C in 95%/5% air/CO_2_.

### 2.2. HEV ORF1 Cloning and Plasmid Constructs

**Figure 1:**
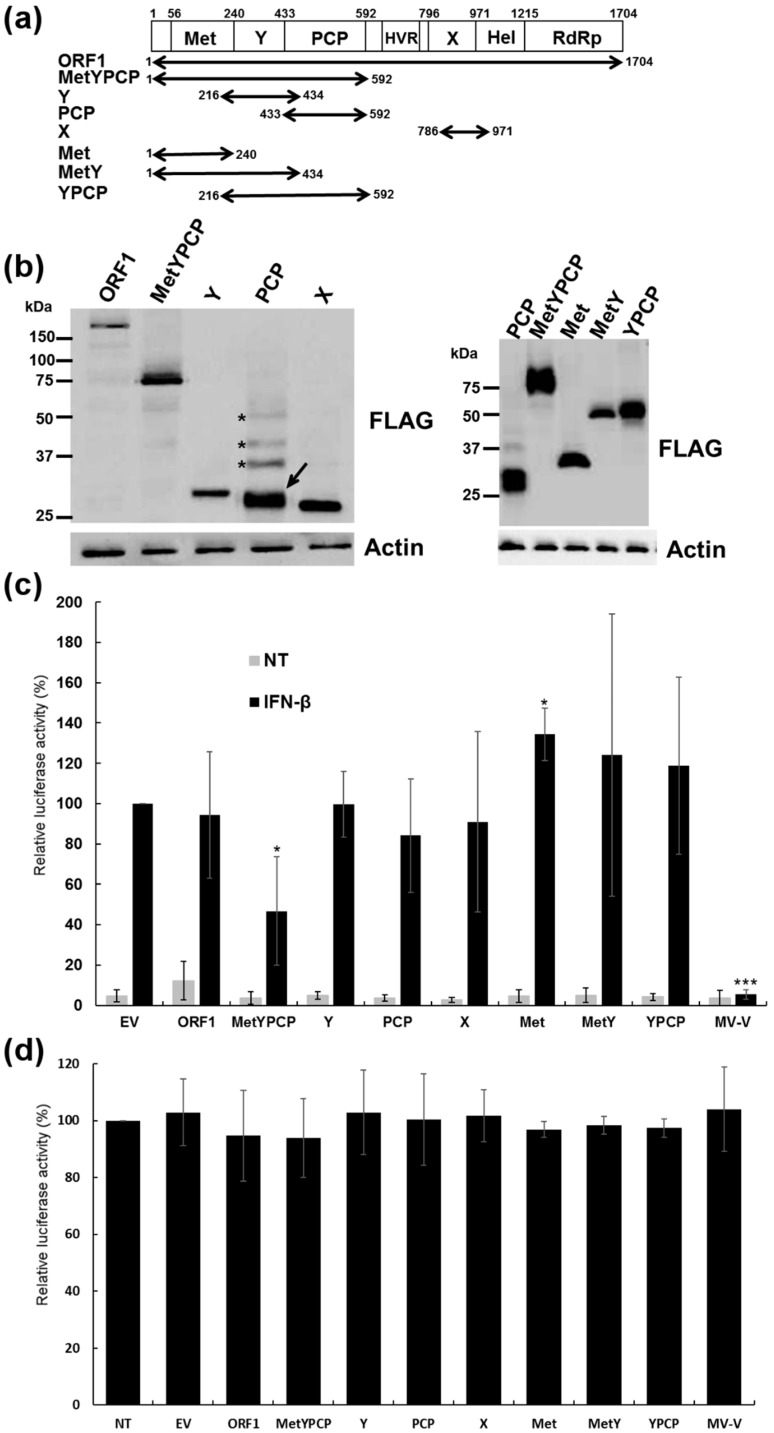
Effect of the expression of full-length HEV ORF1 and several of its domains on IFN-stimulated response element (ISRE) promoter activation. (**a**) Schematic representation of the different domains of HEV ORF1. Met: Methyltransferase domain; Y: Y domain; PCP: Papain-like cysteine protease; HVR: Hypervariable region; X: Macrodomain; Hel: Helicase domain; RdRp: RNA-dependent RNA polymerase. The position of the different putative functional domains present in the ORF1 amino acid sequence of the HEV-3 strain used in this study is indicated. The different fragments of ORF1 that were cloned and expressed in 293T cells are represented by arrows. (**b**) Expression of FLAG-tagged full-length and domains of ORF1 in 293T cells detected by immunoblotting using an anti-FLAG antibody. Bands corresponding to PCP (arrow) and PCP products of higher molecular weight (asterisks) are indicated. Actin served as a loading control. Cells were lysed 18 h post-transfection. (**c**) Effect of full-length ORF1, MetYPCP, Y, PCP, macrodomain (X), Met, MetY and YPCP on ISRE promoter activation: 293T cells were transfected with pISRE-Luc, pCMV-Luc and a pCINeo-3xFLAG empty vector (EV) or a plasmid coding for ORF1, MetYPCP, Y, PCP, X, Met, MetY, YPCP or MV-V. Forty hours later, cells were treated or not (NT) with IFN-β for 7 h and lysed to determine firefly and *Renilla* luciferase activities. Mean ratios between firefly and *Renilla* luciferase activities were calculated and are presented as percentages of the treated EV control (± standard deviations). Results shown represent the mean of four independent experiments performed in triplicate. Here, * *p* < 0.05; *** *p* < 0.0005 compared to EV control for treated samples (unequal variance *t*-tests). Raw data are shown in Supplementary Materials Table S1. (**d**) Cell viability assays at 40 h post-transfection: 293T cells were transfected or not with a pCINeo-3xFLAG empty vector or a plasmid coding for ORF1, MetYPCP, Y, PCP, X, Met, MetY, YPCP or MV-V fused to a 3xFLAG tag. Forty hours after transfection, cells were lysed and cell viability determined using a luminescent-based assay. Luciferase activities (± standard deviations) are expressed as percentage relative to nontransfected cells. No significant difference was found between the cells transfected with the pCINeo-3xFLAG empty vector and the one transfected with the plasmid coding for the different FLAG-tagged proteins (unpaired *t*-tests). Results represent the mean of three independent experiments performed in triplicate.

The serum of a French patient suffering from severe hepatitis E was provided by the former National Reference Centre for HEV (HIA Val de Grâce, Paris, France). A strain of HEV-3f was extracted from this sample using QiAmp viral RNA kit (Qiagen, Hilden, Germany). Reverse transcription-PCR (RT-PCR) was performed with a Primescript reverse transcriptase (Takara Bio Inc., Shiga, Japan). A total of seven overlapping fragments were amplified using the hot start high-fidelity Phusion polymerase (Finnzymes, Thermo Fisher Scientific, Waltham, MA, USA) or a 5′RACE and 3′RACE kit (Invitrogen, Thermo Fisher Scientific, Waltham, MA, USA) and cloned into the plasmid pCR2.1. The 7 overlapping fragments were then digested with restriction enzymes and ligated 2 by 2 with the T4 DNA ligase (Takara Bio Inc., Shiga, Japan) to generate a DNA fragment corresponding to the full-length viral genome downstream of a T7 promoter and a unique SwaI restriction site. This fragment was subsequently cloned into a pUC19 vector to generate pUC19-FR-HuFulHEV3f. The complete nucleotide sequence coding for ORF1 was then determined by sequencing and deposited in the GenBank database under accession number MG197988. The position of the different putative functional domains of ORF1 was identified by comparison with a previous computer-based analysis [3]. DNA sequences coding for full-length ORF1 as well as MetYPCP, Y, PCP, X, Met, MetY and YPCP (Figure 1a) were amplified using pUC19-FR-HuFulHEV3f as a template and specific primers (Table 1) by standard PCR using the Phusion high-fidelity DNA polymerase (Thermo Fisher Scientific, Waltham, MA, USA). The PCR products were then cloned by in vitro recombination into pDONR207 (Gateway system, Invitrogen, Thermo Fisher Scientific, Waltham, MA, USA), as described previously [45]. These coding sequences were subsequently recombined into a translation-optimized pCINeo-3xFLAG expression vector [46] using the Gateway cloning procedure (Invitrogen, Thermo Fisher Scientific, Waltham, MA, USA).

A similar strategy was used to construct the plasmid coding for 3xFLAG-tagged MetYPCP and PCP from HEV-1. RNA from an HEV-1 strain was extracted from a stool sample of a patient with acute hepatitis provided by the previous National Reference Centre for HEV (HIA Val de Grâce, Paris, France) using an RNeasy kit (Qiagen, Hilden, Germany). Reverse transcription was then performed with the PrimeScript Reverse Transcriptase (Takara Bio Inc., Shiga, Japan) according to the manufacturer’s protocol. Three overlapping fragments covering the ORF1 region were amplified using Ex Taq polymerase (Takara Bio Inc., Shiga, Japan) and inserted into TOPO pCR2.1 using the TOPO TA cloning kit (Invitrogen, Thermo Fisher Scientific, Waltham, MA, USA). These 3 constructs were sequenced and used as a template to amplify sequences coding for MetYPCP and PCP with specific primers (Table 1). Expression vectors coding for FLAG-tagged HEV-1 MetYPCP and PCP were then generated using the Gateway cloning procedure (Invitrogen, Thermo Fisher Scientific, Waltham, MA, USA) as described above. The *ORF1* nucleotide sequence of the HEV-1 strain was deposited into the GenBank database under accession number MH976520. The amino acid sequences of the MetYPCP and PCP fragments from this HEV-1 strain were 99% identical to those of the Sar55 HEV-1 strain.

The p3Flag-V plasmid coding for the V protein of a Schwarz strain of measles virus (MV) fused to a 3xFLAG tag [45] was described previously.

### 2.3. Reagents and Antibodies

Recombinant human IFN-β1a was purchased from PBL Interferon Source (Piscataway, NJ, USA), and recombinant human IFN-γ from PeproTech (Rocky Hill, NJ, USA). The mouse anti-actin monoclonal antibody (clone AC-40) and the mouse anti-FLAG (clone M2) were from Sigma-Aldrich (Saint-Louis, MO, USA). Polyclonal antibodies against STAT1 (06-501), phospho-STAT1 (Tyr701) (07-307) and phospho-STAT2 (Tyr 689) (07-224) were from Merck Millipore (Darmstadt, Germany). The rabbit polyclonal antibody against STAT2 (SC-476) was from Santa-Cruz Biotechnology (Dallas, TX, USA).

### 2.4. Transfections

For this, 293T cells were transfected with plasmid DNA using JetPRIME (Polyplus transfection, Strasbourg, France) according to the manufacturer’s instructions.

### 2.5. Cell Viability Test

For this, 293T cells were seeded into a 96-well plate (7.5 × 10^4^ cells/well) and transfected one day later with the different p3xFLAG constructs. Forty hours post-transfection, cells were lysed and cell viability was determined using the CellTiter-Glo^®^ luminescent cell viability assay (Promega, Madison, WI, USA) according to the manufacturer’s recommendations. This assay is based on ATP quantification as an indicator of metabolically active cells.

### 2.6. Immunoblot Analysis

For this, 293T cells were plated in 6-well plates (2 × 10^6^ cells/well) and transfected with 2 μg of the different p3xFLAG constructs. Cells were lysed in radioimmunoprecipitation assay (RIPA) buffer (25 mM Tris HCl (pH 8.8), 50 mM NaCl, 0.5% Nonidet P-40 and 0.1% sodium dodecyl sulphate supplemented with cocktails of protease and phosphatase inhibitors) as previously described [47]. Insoluble material was centrifuged at 16,000× *g* for 20 min at 4 °C and discarded. Total protein concentration of the soluble fraction was determined by Micro BCA^TM^ Protein assay (Pierce, Thermo Fisher Scientific, Waltham, MA, USA). An equal amount of protein extract was reduced by heating in the presence of β-mercaptoethanol and resolved by 12% sodium dodecyl sulphate-polyacrylamide gel electrophoresis (SDS-PAGE) followed by transfer to a nitrocellulose membrane (Hybond-ECL, Amersham, GE healthcare LifeScience, Pittsburgh, PA, USA). Membranes were blocked with phosphate-buffered saline (PBS) containing 5% dry milk and 0.05% Tween-20. Membranes were then incubated with the required dilution of specific antibodies. Bound primary antibodies were detected using horseradish peroxidase-conjugated anti-rabbit or anti-mouse secondary antibodies (Pierce, Thermo Fisher Scientific, Waltham, MA, USA) and an enhanced luminol-based chemiluminescent detection system. Band intensity was measured on scanned immunoblot images using ImageJ software.

### 2.7. Reporter Gene Assay

For this, 293T cells (4 × 10^5^ cells/well) were seeded in 24-well plates. Twenty-four hours later, cells were transfected with 100 ng of firefly luciferase ISRE reporter plasmid containing the ISRE enhancer element upstream of the firefly luciferase gene (pISRE-Luc, Clontech, Mountain View, CA, USA); 10 ng of the reporter plasmid (pCMV-Luc) containing the *Renilla* luciferase gene under the control of the cytomegalovirus (CMV) promoter for normalization of the data; and 250 ng of a plasmid coding for ORF1 or its domains of interest fused to a 3xFLAG tag at their amino-terminal end, or 250 ng of a pCINeo-3xFLAG empty vector as negative control, or 250 ng of a plasmid coding for the V protein of measles virus (MV-V) fused to a 3xFLAG tag as positive control. Forty hours later, the supernatant was removed and replaced with fresh complete medium containing 500 IU/mL of IFN-β. Seven hours later, cells were lysed in passive lysis buffer (Promega, Madison, WI, USA). Firefly and *Renilla* luciferase activity was determined using the Bright-Glo™ luciferase assay system (Promega, Madison, WI, USA) and the Renilla-Glo™ luciferase assay system (Promega, Madison, WI, USA), respectively. The normalized luciferase activity was calculated for each sample by dividing the firefly luciferase activity by the *Renilla* luciferase activity.

### 2.8. RNA Extraction, Reverse Transcription (RT) and Real-Time Quantitative PCR (RT-qPCR)

For this, 293T cells plated in 6-well plates (2 × 10^6^ cells/well) were transfected with 2 μg of a pCINeo-3xFLAG empty vector or a plasmid coding for MetYPCP, PCP or MV-V fused to a 3xFLAG tag. Forty hours post-transfection, cells were stimulated for 6 h with 500 IU/mL of IFN-β. Total RNA was extracted using the RNeasy minikit (Qiagen, Hilden, Germany), including a digestion step on columns with DNase I (Qiagen, Hilden, Germany). A second digestion step was performed using a TURBO DNase (Ambion, Thermo Fisher Scientific, Waltham, MA, USA), and the RNA was cleaned up on a column using the RNeasy minikit (Qiagen, Hilden, Germany). RT was done using 500 ng of RNA with PrimeScript Reverse Transcriptase (Takara Bio Inc., Shiga, Japan) according to the manufacturer’s instruction. RT-qPCR was performed on 2 µL of cDNA using the SYBR Green Master Mix kit (Roche, Basel, Switzerland) and specific primers (Table 1). A LightCycler 96 apparatus (Roche, Basel, Switzerland) was used for sample analysis. Samples were denatured for 15 min at 95 °C, and then DNA was amplified for 40 cycles at 95 °C for 30 s, 60 °C for 30 s and 72 °C for 30 s. The final extension was followed by cooling at 40 °C for 30 s. Relative quantification was realized using the 2-*ΔΔ*C_T_ method [48]. Glyceraldehyde 3-phosphate dehydrogenase (GAPDH) was used as an endogenous control for normalization. The mean *Δ*C_T_ obtained in nonstimulated cells transfected with the empty vector was used as the calibrator.

### 2.9. Immunostaining and Fluorescent Microscopy

For this, 293T cells (3.5 × 10^5^ cells/well) were seeded onto 12-mm-diameter coverslips previously coated with poly-d-lysine (Sigma-Aldrich, Saint-Louis, MO, USA) in 24-well plates and transfected with 250 ng of a pCINeo-3xFLAG empty vector (EV) or a plasmid coding for ORF1, MetYPCP, PCP, X, Y or MV-V fused to a 3xFLAG tag. Twenty-four hours later, cells were treated or not with IFN-β or –γ for 30 min, washed with PBS and fixed with 4% paraformaldehyde in PBS. Cells were permeabilised with 0.2% Triton X-100 in PBS and incubated in blocking buffer (0.5% BSA in PBS). The appropriate dilution of primary antibodies was then added for 1 h at room temperature. Cells were then washed several times in PBS, and DyLight^TM^ 488 anti-mouse and DyLight^TM^ 550 anti-rabbit secondary antibodies (Thermo Scientific, Thermo Fisher Scientific, Waltham, MA, USA) were used to detect bound primary antibodies. Samples were mounted in Mowiol containing 4,6-diamidine-2-phenylindole dihydrochloride (DAPI) (Sigma-Aldrich, Saint-Louis, MO, USA). Microscopy was carried out with an Axio observer Z1 fluorescent microscope (Zeiss, Oberkochen, Germany), and images were acquired using Zen 2012 software.

### 2.10. Statistical Analyses

An unpaired *t*-test or an unequal variance *t*-test were used to analyse the data. Differences were considered to be significant if the *p* value was <0.05.

## 3. Results

### 3.1. Expression of Full-Length and Individual Domains of HEV ORF1

Sequences coding for full-length ORF1 and the MetYPCP, Y, PCP and X domains of an HEV-3f strain were identified according to a previous computer-based analysis [3], amplified and inserted in an expression vector downstream and in frame of a sequence coding for a 3xFLAG tag (Figure 1a). Expression of the different constructs was confirmed in 293T cells by immunoblotting (Figure 1b). These human embryonic kidney cells were used, as they give high transfection efficiency that cannot be reached in hepatic cell lines. Bands corresponding to the expected molecular weight of FLAG-ORF1 (192 kDa), FLAG-Y (31 kDa), FLAG-X (26 kDa) and FLAG-MetYPCP (72 kDa) were detected. Bands of lower molecular weight were also observed for FLAG-ORF1 and FLAG-MetYPCP, suggesting cleavage or degradation of these proteins. In contrast, bands corresponding to higher molecular weights than the one expected (23 kDa) were detected for FLAG-PCP, suggesting post-translational modification or dimerization of the viral protein (Figure 1b).

### 3.2. MetYPCP of HEV ORF1 Inhibited the IFN-I Response

To assess the ability of the different HEV ORF1 products to interfere with the IFN-I response, we first examined their effect on ISRE promoter activation using a luciferase reporter assay. For this, 293T cells were transfected with an ISRE-reporter plasmid (pISRE-Luc), a control vector (pCMV-Luc) to normalize for transfection and a pCINeo-3xFLAG empty vector or plasmids coding for ORF1, MetYPCP, Y, PCP or X. Cells were treated 40 h later with IFN-β for 7 h. As a positive control, cells were transfected with a plasmid coding for the V protein of the Schwarz strain of measles virus (MV-V) fused to a FLAG tag at its amino-terminal. This viral protein inhibits the IFN-I response by interacting with STAT1 and JAK1 and interfering with STAT1 and TYK2 phosphorylation [45]. As shown in Figure 1c and Supplementary Materials Table S1, the expression of MetYPCP was able to inhibit significantly ISRE promoter activation after stimulation with IFN-β. This inhibition was not due to a cytotoxic effect of the viral protein, as transfection of the construct coding for MetYPCP for 40 h did not affect cell viability (Figure 1d). The MetYPCP inhibitory effect on the ISRE promoter was modest compared to the one of MV-V. This might have been due to the low expression level of MetYPCP in our reporter assay (Supplementary Materials Figure S1). In contrast, no significant inhibition was detected when PCP alone was expressed. It is interesting to note that both MetYPCP and PCP were able to inhibit significantly IFN-β promoter activity after stimulation of the RLR pathway in a luciferase reporter assay [49]. This result was in agreement with previous findings showing that PCP from HEV-1 ORF1 was an inhibitor of the RLR pathway [35] and suggested that the PCP domain expressed in our study was functional. ORF1 had no impact on ISRE promoter activation in our assay, but the relatively low expression of full-length ORF1 or a lack of processing of the polyprotein in 293T cells (Figure 1b and Supplementary Materials Figure S1) might have masked a putative inhibitory effect. To determine whether the entire MetYPCP product was necessary to inhibit ISRE promoter activation, we also tested the effect of Met, MetY and YPCP of HEV ORF1 on ISRE promoter activity using the same luciferase reporter assay (Figure 1a–c, Supplementary Materials Figure S1 and Table S1). As shown in Figure 1c, expression of Met alone resulted in a significant increase in ISRE promoter activation, and MetY or YPCP had no effect on ISRE promoter activation. These results suggested that expression of the amino-terminal region of ORF1 containing the Met, Y and PCP domains was necessary to inhibit the signalling pathway triggered by IFN-I.

**Figure 2:**
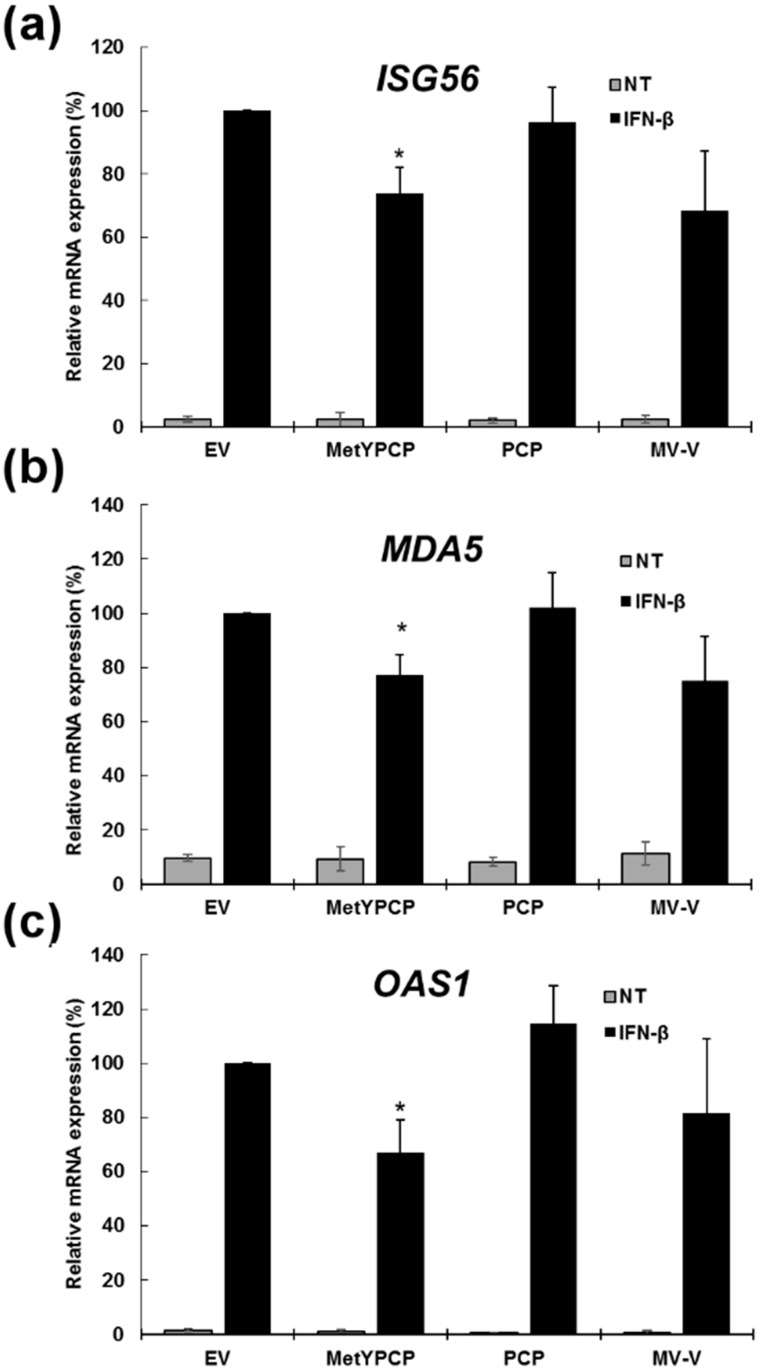
Expression of MetYPCP of HEV ORF1 downregulated mRNA levels of several ISGs following IFN-β treatment. (**a**–**c**) Here, 293T cells were transfected with a pCINeo-3xFLAG empty vector or a plasmid coding for MetYPCP, PCP or MV-V fused to a 3xFLAG tag. Forty hours post-transfection, cells were stimulated or not (NT) with 500 IU/mL of IFN-β for 6 h. Total RNA was extracted, and expression of the mRNA coding for (**a**) ISG56, (**b**) melanoma differentiation-associated protein (MDA)5 and (**c**) 2′,5′-oligoadenylate synthetase (OAS)1 were measured by RT-qPCR. *Glyceraldehyde 3-phosphate dehydrogenase* (*GAPDH*) was used as a reference gene. Relative mRNA expression was calculated for each condition and is presented as percentages of the treated EV control (± standard deviations). Results shown represent the mean of three independent experiments performed in triplicate: *, *p* < 0.05 compared to EV control for treated samples (unequal variance *t*-tests). Results from the three independent experiments are presented in Supplementary Materials Figure S2.

We then focussed on MetYPCP. To further confirm its antagonistic effect on the IFN-I response, we examined the level of induction of 3 ISG mRNAs after IFN-β treatment by RT-qPCR upon MetYPCP expression (Figure 2 and Supplementary Materials Figure S2). We also included in our assay MV-V (a positive control) and PCP (as an additional negative control), as this domain had no effect on ISRE promoter activation (Figure 1c): 293T cells were transfected with an empty vector or a plasmid coding for MV-V, PCP or MetYPCP for 40 h before stimulation with IFN-β for 6 h. We found that, following IFN-β treatment, expression of MetYPCP was able to significantly downregulate the mRNA levels of ISG56 (Figure 2a), melanoma differentiation-associated protein 5 (MDA5) (Figure 2b) and 2′,5′-oligoadenylate synthetase 1 (OAS1) (Figure 2c). These results confirmed our previous observation (Figure 1c) that MetYPCP, but not PCP alone, was able to counteract the IFN-I response.

### 3.3. MetYPCP of HEV ORF1 Interfered with the JAK/STAT Pathway after IFN-β Treatment

**Figure 3:**
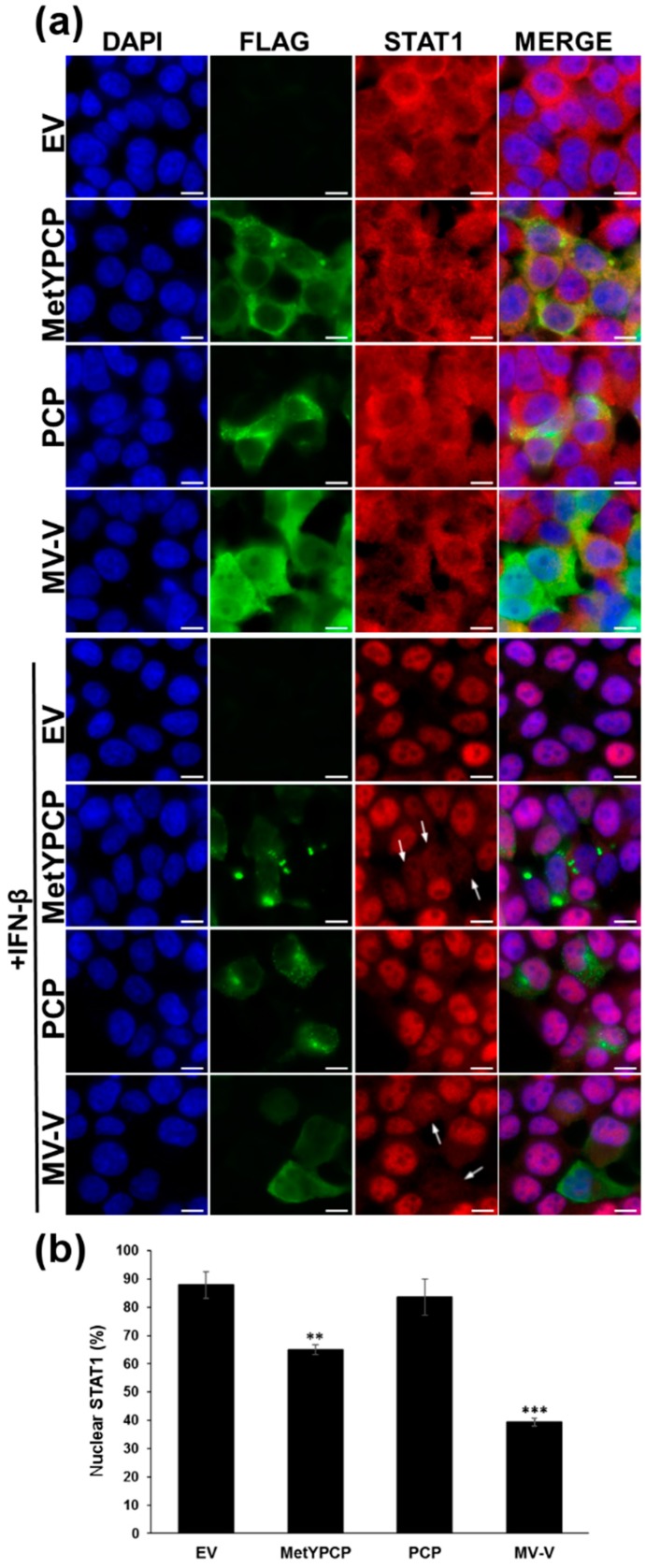
Expression of MetYPCP of HEV ORF1 decreased signal transducer and activator of transcription protein (STAT)1 nuclear translocation upon IFN-β treatment. (**a**) Here, 293T cells were transfected with a pCINeo-3xFLAG empty vector or a plasmid coding for MetYPCP, PCP or MV-V fused to a 3xFLAG tag. Twenty-four hours post-transfection, cells were stimulated or not for 30 min with 1000 IU/mL of IFN-β. Cells were then washed, fixed and stained with primary antibodies raised against STAT1 and FLAG, followed by fluorescent dye-conjugated secondary antibodies. Intracellular localization of 4,6-diamidine-2-phenylindole dihydrochloride (DAPI)-stained nuclei (blue), FLAG (green) and STAT1 (red) was visualized by microscopy (magnification, 630×). Scale bars: 10 μm. Cells showing diffuse cytoplasmic/nuclear localisation of STAT1 upon IFN-β treatment are indicated by arrows. (**b**) STAT1 localization was visualized after immunostaining as described in (**a**) in 293T cells transfected with a pCINeo-3xFLAG empty vector or a plasmid coding for MetYPCP, PCP or MV-V fused to a FLAG tag. For each condition, STAT1 localisation (predominant nuclear localisation or diffuse localisation within the cytoplasm and nucleus) was determined in 70 to 172 cells expressing the corresponding FLAG-tagged protein (except for the EV control, for which 356 to 384 cells were randomly assessed). The mean percentage (± standard deviation) of cells showing a predominant nuclear localization of STAT1 from three independent experiments is shown: ** *p* < 0.005; *** *p* < 0.0005 compared to EV control for treated samples (unpaired *t*-tests).

To better understand the mechanisms involved in the inhibition of the IFN-I response by MetYPCP, we examined whether the viral protein was able to interfere with the JAK/STAT pathway. First, we assessed the ability of MetYPCP to modulate STAT1 nuclear translocation after IFN-β stimulation by immunofluorescence. In the absence of IFN treatment, STAT1 was found predominantly in the cytoplasm of 293T cells transfected with an empty vector or plasmids coding for FLAG-tagged MetYPCP, PCP or MV-V (Figure 3a, upper panels). STAT1 was also found in the nucleus as the transcription factor shuttled between the nucleus and the cytoplasm in unstimulated cells [50]. IFN-β treatment led to the nuclear accumulation of STAT1 in most of the cells transfected with an empty vector or a plasmid coding for PCP (Figure 3a, lower panels). In contrast, STAT1 remained diffuse in the cytoplasm and nucleus of a proportion of cells expressing MetYPCP and MV-V (Figure 3a, lower panels). We then quantified the number of cells showing predominant nuclear localisation and the number of cells with a cytoplasmic/nuclear localisation of STAT1 upon IFN-β stimulation (Figure 3b). A significant decrease in the percentage of cells showing STAT1 nuclear localisation was found in cells expressing MetYPCP (65%) and MV-V (39%) in comparison to cells transfected with the empty plasmid (88%) or expressing PCP (84%), thus suggesting that MetYPCP interfered with STAT1 nuclear translocation. In addition, ORF1, Met, Y and X had no effect on STAT1 nuclear translocation (Supplementary Materials Figure S3), thus corroborating our results from the reporter assay (Figure 1c). Interestingly, MetYPCP localised more frequently in dense structures around the nucleus upon IFN-β stimulation. It is possible that the viral protein interacted with a cellular protein whose expression was induced by IFN-β, leading to a change in its localisation upon stimulation. This difference of localisation was also observed with PCP but was less apparent than for MetYPCP.

### 3.4. MetYPCP of HEV ORF1 Inhibited STAT1 but Not STAT2 Phosphorylation after IFN-β Treatment

**Figure 4:**
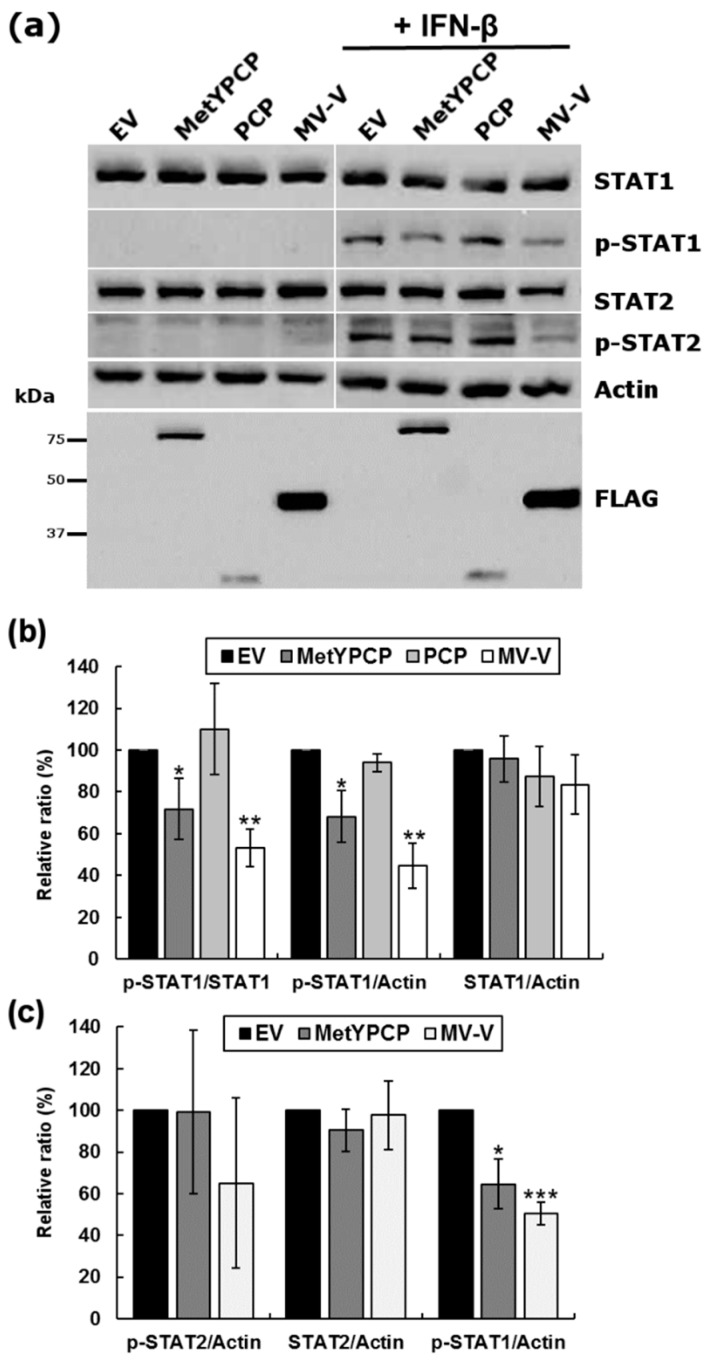
Expression of MetYPCP of HEV ORF1 inhibited STAT1 but not STAT2 phosphorylation upon IFN-β treatment. (**a**) Here, 293T cells were transfected with a pCINeo-3xFLAG empty vector or a plasmid coding for MetYPCP, PCP or MV-V fused to a 3xFLAG tag. Twenty-four hours post-transfection, cells were stimulated for 30 min with 500 IU/mL of IFN-β. Cell lysates were extracted and used for the detection of FLAG-tagged proteins, total STAT1, phosphorylated STAT1 (p-STAT1), total STAT2 and phosphorylated STAT2 (p-STAT2) by immunoblotting. Actin served as an internal control. (**b**) Here, 293T cells were transfected with an empty vector or a plasmid coding for MetYPCP, PCP or MV-V and treated as described in (**a**). Cell lysates were extracted and used for the detection of total STAT1, p-STAT1 and actin by immunoblotting. Band intensities were quantified using ImageJ software, and relative levels of STAT1, p-STAT1 and actin were determined for each treated sample. Ratios between p-STAT1 and actin, STAT1 and actin, and p-STAT1 and STAT1 were calculated and expressed as a relative percentage in comparison to the EV control. (**c**) Here, 293T cells were transfected with an empty vector or a plasmid coding for MetYPCP or MV-V and treated as described in (**a**). Cell lysates were extracted and used for the detection of total STAT2, p-STAT2 and p-STAT1 by immunoblotting. Band intensities were quantified using ImageJ software, and relative levels of STAT2, p-STAT2, p-STAT1 and actin were determined for each treated sample. Ratios between p-STAT2 and actin and STAT2 and actin were calculated and expressed as a relative percentage in comparison to the EV control. The ratio between p-STAT1 and actin was also determined to ensure significant inhibition of the p-STAT1 level by MetYPCP in this set of experiments. In (**b**–**c**), the mean percentage (± standard deviation) of four independent experiments is presented for each panel: * *p* < 0.05; ** *p* < 0.005; *** *p* < 0.0005 compared to the EV control for IFN-treated samples (unequal variance *t*-tests).

To investigate which step of the JAK/STAT pathway was targeted by MetYPCP, we then assessed the phosphorylation status of STAT1 after IFN-β treatment by immunoblot analysis in 293T cells expressing MetYPCP or PCP. Cells expressing MV-V were used as positive controls. As shown in Figure 4a,b, the level of phosphorylated STAT1 detected after IFN-β treatment was reduced significantly in 293T cells expressing MetYPCP in comparison to cells transfected with an empty vector or expressing PCP. No change in the total level of STAT1 was observed in cells expressing MetYPCP, indicating that the viral protein did not interfere with the expression or stability of STAT1. Moreover, we also assessed the phosphorylation status of STAT2 and found that MetYPCP had no significant effect on the level of total and phosphorylated STAT2 (Figure 4a,c), suggesting that MetYPCP interfered with the activation of STAT1 but not STAT2 following IFN-I treatment.

### 3.5. MetYPCP of HEV ORF1 Inhibited More Efficiently the JAK/STAT Pathway after IFN-I Than IFN-II Treatment

**Figure 5:**
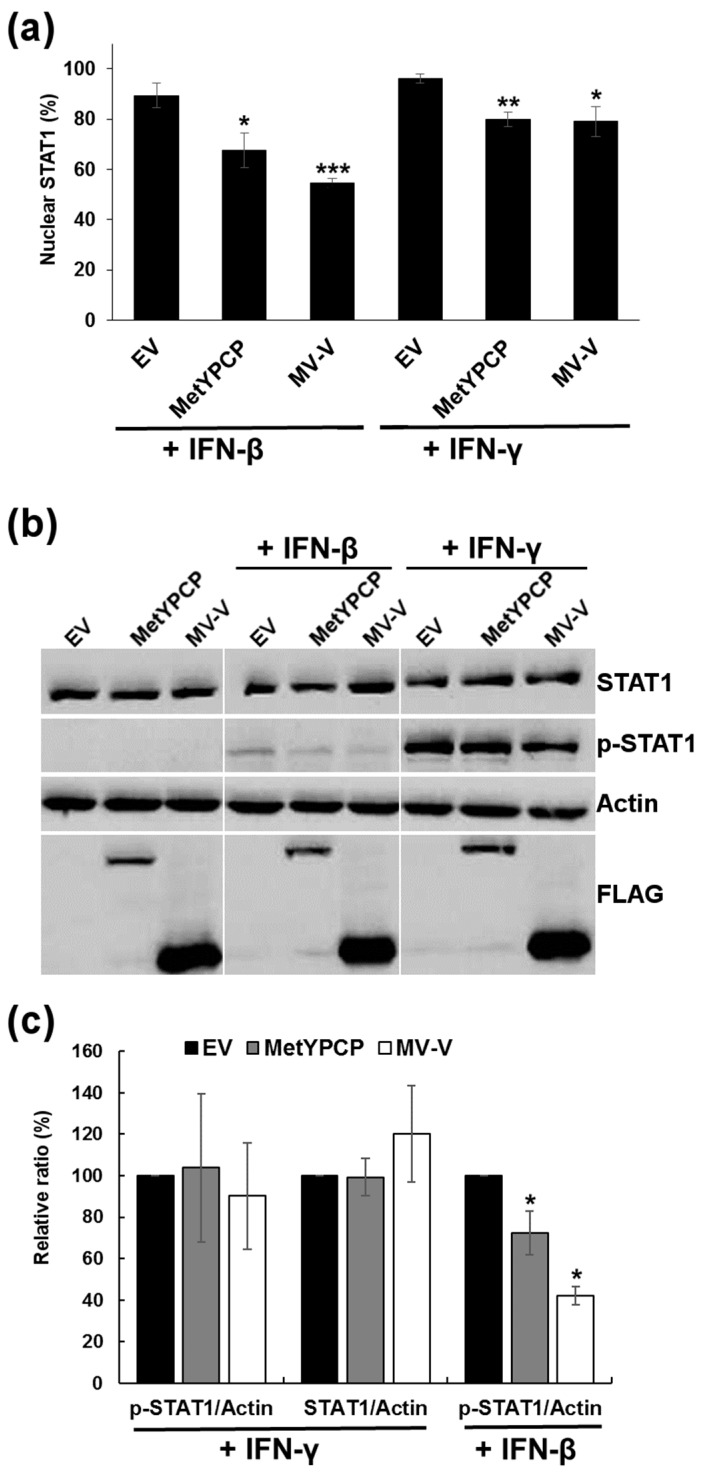
Expression of MetYPCP of HEV ORF1 inhibited weakly STAT1 translocation but not STAT1 phosphorylation in response to IFN-II. (**a**) Here, 293T cells were transfected with a pCINeo-3xFLAG empty vector or a plasmid coding for MetYPCP or MV-V fused to a 3xFLAG tag. Twenty-four hours post-transfection, cells were stimulated for 30 min with 1000 IU/mL of IFN-β or 250 ng/mL of IFN-γ. Cells were then washed, fixed and stained with primary antibodies raised against STAT1 and FLAG, followed by fluorescent dye-conjugated secondary antibodies. STAT1 localization was determined in 64 to 102 cells expressing the corresponding FLAG-tagged protein (except for the EV control, for which 299 to 328 cells were randomly assessed). The mean percentage (± standard deviation) of cells showing a predominant nuclear localization of STAT1 from three independent experiments is shown: * *p* < 0.05; ** *p* < 0.005; *** *p* < 0.0005 compared to the EV control for treated samples (unpaired *t*-tests). (**b**) Here, 293T cells were transfected with a pCINeo-3xFLAG empty vector or a plasmid coding for MetYPCP, PCP or MV-V fused to a 3xFLAG tag. Twenty-four hours post-transfection, cells were stimulated for 30 min with 500 IU/mL of IFN-β or 250 ng/mL of IFN-γ. Cell lysates were extracted and used for the detection of FLAG-tagged proteins, total STAT1, phosphorylated STAT1 (p-STAT1) and actin as an internal control by immunoblotting. (**c**) Band intensities were quantified using ImageJ software, and relative levels of STAT1, p-STAT1 and actin were determined for each sample treated with 125 or 250 ng/mL of IFN-γ or 500 IU/mL of IFN-β. Ratios between p-STAT1 and actin and STAT1 and actin were then calculated and expressed as a relative percentage in comparison to the EV control. The mean percentage (± standard deviation) of three independent experiments is presented: * *p* < 0.05 compared to the EV control (unequal variance *t*-tests).

We then wanted to determine whether MetYPCP had the ability to inhibit the JAK/STAT pathway in response to IFN-II. As IFN-I and -II activation triggers different components of the JAK/STAT pathway, these experiments could help pinpoint at which level of the pathway MetYPCP was acting. First, we assessed the effect of MetYPCP expression on STAT1 nuclear translocation after IFN-γ stimulation by immunofluorescence (Figure 5a and Supplementary Materials Figure S4). IFN-γ treatment led to the nuclear translocation of STAT1 in around 96% of cells transfected with an empty vector and around 80% of cells expressing MetYPCP (Figure 5a), thus suggesting that MetYPCP was able to inhibit STAT1 translocation in response to IFN-γ. However, this antagonist effect was less pronounced than the one observed after IFN-β treatment, for which 68% of cells expressing MetYPCP displayed a predominant localization of STAT1 in the nucleus (Figure 5a). We also assessed the ability of MetYPCP to inhibit STAT1 phosphorylation after IFN-II treatment. Cells expressing MetYPCP were treated with IFN-γ for 30 min, and the level of phosphorylated STAT1 was quantified by immunoblotting. As shown in Figure 5b,c, no significant inhibition of STAT1 phosphorylation was detected in cells expressing MetYPCP following IFN-γ treatment. These results suggested that MetYPCP had a limited impact on the JAK/STAT pathway after IFN-II treatment. Similarly to MetYPCP, MV-V caused a slight decrease of STAT1 translocation (Figure 5a) and did not inhibit STAT1 phosphorylation (Figure 5b,c) after IFN-γ treatment. These results were in agreement with several studies showing that MV-V was more efficient at antagonizing the response to IFN-I in comparison to IFN-II [51,52,53].

### 3.6. The Ability of MetYPCP of HEV ORF1 to Inhibit the JAK/STAT Pathway after IFN-I Differed between Genotypes

**Figure 6:**
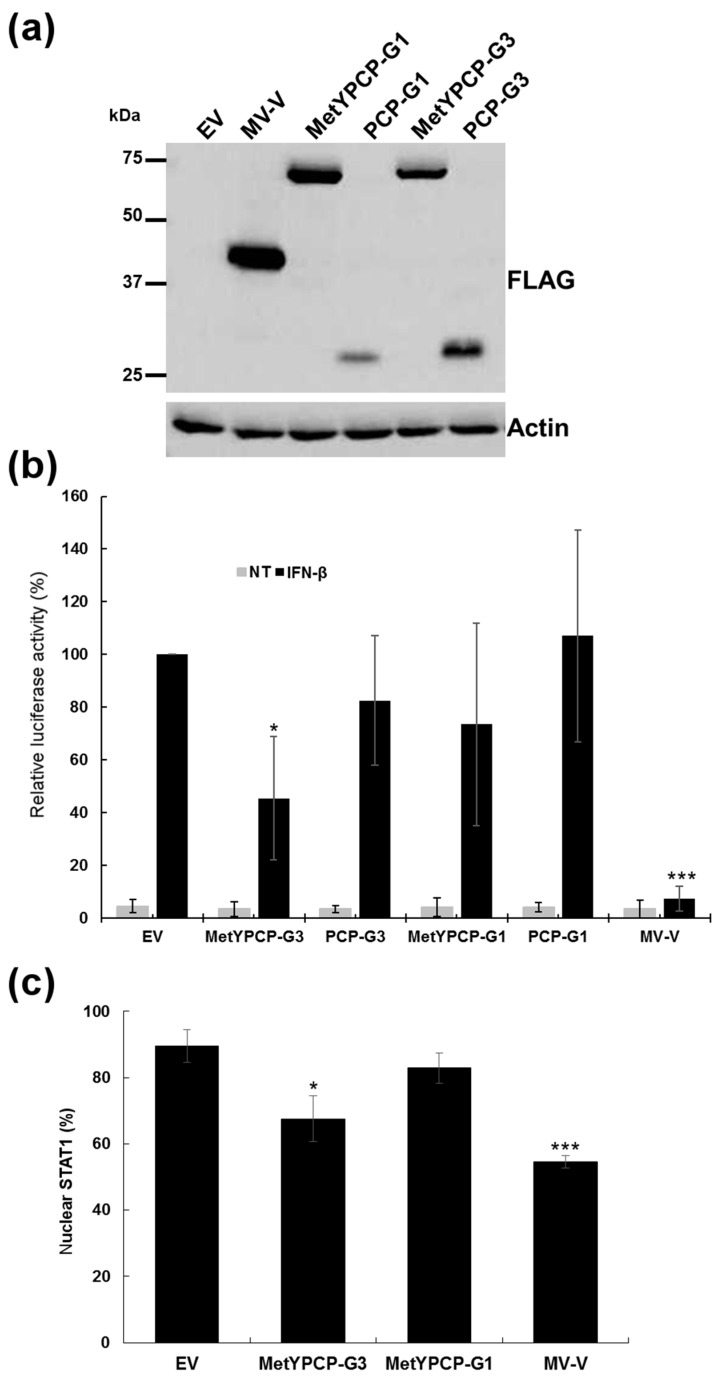
Comparison of the effect of MetYPCP from HEV-1 and HEV-3 on the Janus kinase (JAK)/STAT pathway. (**a**) Expression of FLAG-tagged MetYPCP and PCP from a strain of HEV-1 (MetYPCP-G1 and PCP-G1) and HEV-3 (MetYPCP-G3 and PCP-G3) in 293T cells detected by immunoblotting using an anti-FLAG antibody. Actin served as a loading control. Cells were lysed 24 h post-transfection. (**b**) Effect of MetYPCP and PCP from HEV-1 and HEV-3 on ISRE promoter activation: 293T cells were transfected with pISRE-Luc, pCMV-Luc and a pCINeo-3xFLAG empty vector or a plasmid coding for MV-V, MetYPCP-G1, MetYPCP-G3, PCP-G1 or PCP-G3. Forty h later, cells were treated or not (NT) with IFN-β for 7 h and lysed to determine firefly and *Renilla* luciferase activities. Mean ratios between firefly and *Renilla* luciferase activities were calculated and are presented as percentages of the treated EV control (± standard deviations). Results shown represent the mean of five independent experiments performed in triplicate: * *p* < 0.05; *** *p* < 0.0005 compared to the EV control for treated samples (unequal variance *t*-tests). Raw data are presented in Supplementary Materials Table S2. (**c**) Here, 293T cells were transfected with a pCINeo-3xFLAG empty vector or a plasmid coding for MetYPCP-G3, MetYPCP-G1 or MV-V fused to a 3xFLAG tag. Twenty-four hours post-transfection, cells were stimulated for 30 min with 1000 IU/mL of IFN-β. Cells were then washed, fixed and stained with primary antibodies raised against STAT1 and FLAG, followed by fluorescent dye-conjugated secondary antibodies. STAT1 localization was determined in 70 to 117 cells expressing the corresponding FLAG-tagged protein (except for the EV control, for which 311 to 328 cells were randomly assessed). The mean percentage (± standard deviation) of cells showing a predominant nuclear localization of STAT1 from three independent experiments is shown: * *p* < 0.05; *** *p* < 0.0005 compared to the EV control for treated samples (unpaired *t*-tests).

We then wondered whether the ability of MetYPCP to inhibit the JAK/STAT pathway was genotype-specific and differed between “human only” (HEV-1) and zoonotic (HEV-3) genotypes. To achieve this, the sequences coding for the MetYPCP and PCP domains of an HEV-1 strain were cloned and inserted into a 3xFLAG expression vector. We found an amino acid sequence identity of 86% between the MetYPCP domains from the HEV-1 and HEV-3 strains cloned in this study (Supplementary Materials Figure S5) and of 69% between the PCP domains. Expression of the ORF1 fragments was then confirmed in 293T cells by immunoblotting (Figure 6a). The effect of the HEV-1 MetYPCP and PCP domains on ISRE promoter activation was then assessed using the luciferase reporter assay described above. As shown in Figure 6b and Supplementary Materials Table S2, MetYPCP from HEV-3, but not HEV-1, was able to inhibit ISRE promoter activation after IFN-β treatment. This difference was not due to a problem of expression of HEV-1 MetYPCP, as this domain was more efficiently expressed in 293T cells than HEV-3 MetYPCP and did not impact cell viability (Figure 6a, Supplementary Materials Figures S6 and S7). In agreement with this result, we also found that the expression of MetYPCP from HEV-3, but not HEV-1, inhibited significantly STAT1 nuclear translocation after IFN-β stimulation (Figure 6c and Supplementary Materials Figure S8). Altogether, these results suggested that the MetYPCP domain from HEV-1 was not able to inhibit the JAK/STAT pathway as efficiently as the one from HEV-3 and that differences in the ability of MetYPCP to interfere with the JAK/STAT pathway existed between HEV genotypes.

## 4. Discussion

Most viruses encode multifunctional viral proteins that counteract the host antiviral response at several levels of the IFN induction and signalling pathways [54]. Recent studies have reported that the PCP domain, the macrodomain and the Met domain of HEV ORF1 were antagonists of IFN induction [35,36]. The macrodomain was shown to interfere with IRF-3 phosphorylation, whereas PCP was able to deubiquitinate components of the RLR pathway such as RIG-I and TANK binding kinase 1 (TBK-1) in 293T cells [35]. Here, we showed that the amino-terminal region of HEV ORF1 was able to inhibit the IFN-I response by targeting the JAK/STAT pathway. Thus, domains of the nonstructural polyprotein ORF1 counteracted the host IFN system at the level of IFN induction [35,36] and IFN signalling (our study).

In this study, we overexpressed the tagged version of ORF1 and several of its domains in 293T cells to study their potential inhibitory functions on the IFN-I response. This strategy allowed us to overcome the difficulties encountered to efficiently infect cells in vitro with HEV and detect ORF1 protein expression [10,37]. In future studies, it will be important to validate the effect of MetYPCP on the JAK/STAT pathway in hepatocytes, the main targets of the virus. Nevertheless, several studies have suggested that HEV replicates in the kidney, as HEV RNA and/or antigens have been detected in the kidney tissues of HEV-infected gerbils [55], monkeys [56], swine [57] and rabbits [58,59]. Being embryonic kidney cells, 293T cells thus represent a relevant model to study HEV.

We found that a protein encompassing the predicted Met, Y and PCP domains of HEV ORF1 inhibited ISRE promoter activation and the expression of several ISGs in response to IFN-β. Further investigations revealed that MetYPCP interfered with IFN-β-induced STAT1 nuclear translocation and phosphorylation, thus indicating that MetYPCP targeted the JAK/STAT pathway. Moreover, MetYPCP seemed to act specifically on STAT1 activation, as STAT2 phosphorylation was not affected by the expression of this ORF1 product. STAT1 is a key component of the JAK/STAT pathway that is targeted by a large number of viral proteins, and multiple mechanisms of inhibition have been described [54,60]. Some viral proteins interact directly with STAT1 to block its phosphorylation, while others act as phosphatase to dephosphorylate STAT1 or sequester STAT1 in the cytoplasm or induce its degradation [54,60]. Here, we found that MetYPCP did not affect total levels of STAT1, suggesting that MetYPCP was not able to degrade the cellular protein or affect its expression. However, we found that MetYPCP was able to inhibit STAT1 phosphorylation more efficiently in response to IFN-β than to IFN-γ, thus suggesting that MetYPCP interfered more specifically with one or several components or regulators of the JAK/STAT pathway triggered by IFN-I. Activation of the JAK/STAT pathway by type II IFN involves a specific receptor (IFNGR) and the phosphorylation of JAK1, JAK2 and STAT1, but not TYK2 and STAT2, which are activated by IFN-I only. One can then hypothesize that MetYPCP interferes with the recruitment of STAT1 to the IFNAR subunits or with the phosphorylation of STAT1 by TYK2. MetYPCP could also interfere with cellular proteins involved in the regulation of IFN-I-driven STAT1 phosphorylation. In addition, it is possible that MetYPCP targets several steps of the JAK/STAT pathway and that one target (upstream STAT1 phosphorylation) is specific to the IFN-I response while another (upstream STAT1 translocation) is common to both the IFN-I and -II response. This would explain why we found that MetYPCP inhibited significantly the translocation of STAT1 but not its phosphorylation after IFN-II treatment.

We also found that only the ORF1 product containing the predicted functional Met, Y and PCP domains was able to inhibit ISRE promoter activation, and not Met, Y or PCP alone or the combination of Met and Y or Y and PCP. Surprisingly, in contrast to MetYPCP, expression of Met significantly enhanced ISRE promoter activation. We did not investigate further the mechanism involved in this stimulatory effect. The function of Met might differ depending on whether it is expressed alone or coexpressed with other ORF1 domains. One region of MetYPCP might be needed for its localisation or its catalytic activity while another interacts with specific cellular protein(s). A previous study has reported that a putative zinc-finger domain is present in Met (between amino acids 73 and 94) that might be critical for the enzymatic activity of PCP [44]. Many viral cysteine proteases require a zinc-binding finger motif to be catalytically active or to function as an antagonist of the IFN response. For example, the zinc-finger domain of Nsp1-α of porcine reproductive and respiratory syndrome virus (PRRSV) is critical for the viral protein to inhibit IFN-β synthesis [61]. One can envisage that the enzymatic activity of HEV PCP is dependent on a zinc-finger domain present in Met and is important for the inhibitory action of MetYPCP. This would then explain why MetYPCP was able to inhibit the JAK/STAT pathway, while PCP alone did not, or did less efficiently. In future work, it would be interesting to assess the inhibitory effect of MetYPCP mutants with a disrupted zinc-binding finger motif to check this hypothesis.

Our results showed that the ability of MetYPCP to inhibit the JAK/STAT pathway differed according to the HEV genotype involved. This result needs to be further investigated, as this difference could explain, at least partially, why distinct pathogenesis and species tropisms are observed between “human only” (HEV-1) and zoonotic (HEV-3) genotypes. The amino acid sequence of MetYPCP from genotype 1 is 86% identical to that of MetYPCP from genotype 3 (Supplementary Materials Figure S5). The main differences in the amino acid sequence between the two genotypes are present in the C-terminal part (amino acids 454–592), which corresponds to the PCP domain (433–592). It would then be interesting to construct MetYPCP chimeras carrying the MetY domain from HEV-3 and the PCP domain from HEV-1 and vice versa to determine which part of MetYPCP is responsible for its inhibitory function on the JAK/STAT pathway. In addition, a recent paper has suggested that a factor Xa cleavage site is present at amino acid 560 within the PCP domain of HEV-1 strains but is not present in HEV-3 strains [12]. Such differences in the processing of ORF1 between genotypes could affect its function as an IFN-I antagonist and needs to be better characterised.

## 5. Conclusions

Until recently, very few studies have been undertaken to understand how HEV interacts with the immune system of its host. Data from this study expanded our knowledge on the mechanisms evolved by HEV to counteract the IFN response and provided additional evidence that ORF1 plays multiple roles in this evasion strategy. A better understanding of the signalling pathways targeted by HEV proteins to modulate the host antiviral response will help to identify new therapeutic targets and to improve the prevention and control of HEV infection. This is critical, as no anti-HEV drug has been approved yet, and this will be particularly relevant for the treatment of chronic cases of hepatitis E in immunosuppressed patients.

## Figures and Tables

**Table 1:** Primers used for the amplification of DNA sequences coding for full-length or fragments of hepatitis E virus (HEV)-3 and HEV-1 open reading frame (ORF)1 and for the quantification of interferon (IFN)-stimulated gene (ISG) expression by real-time quantitative PCR (RT-qPCR). F: forward primer; R: reverse primer.

Gene Product	Primers
***ORF1***	**F:** GGGGACAACTTTGTACAAAAAAGTTGGCATGGAGGCCCACCAGTTCATT **R:** GGGGACAACTTTGTACAAGAAAGTTGGTCATTCCAACCTCTGTATGAT
***Met***	**F:** GGGGACAACTTTGTACAAAAAAGTTGGCATGGAGGCCCACCAGTTCATT **R:** GGGGACAACTTTGTACAAGAAAGTTGGTTAGATCCATGCACGAAGTATAG
***Y***	**F:** GGGGACAACTTTGTACAAAAAAGTTGGCCGCGCCGTCGTGACTTATGAG **R:** GGGGACAACTTTGTACAAGAAAGTTGGTTAGCACTGTGCATAAAACTGTAG
***PCP***	**F:** GGGGACAACTTTGTACAAAAAAGTTGGCCAGTGCCGCCGCTGGCTCTCA **R:** GGGGACAACTTTGTACAAGAAAGTTGGTTACAAAACATACTGTTCGGGACCGTTG
***MetYPCP***	**F:** GGGGACAACTTTGTACAAAAAAGTTGGCATGGAGGCCCACCAGTTCATT **R:** GGGGACAACTTTGTACAAGAAAGTTGGTTACAAAACATACTGTTCGGGACCGTTG
***X***	**F:** GGGGACAACTTTGTACAAAAAAGTTGGCGCCCGCACTCGCCGGCTCCTT **R:** GGGGACAACTTTGTACAAGAAAGTTGGTTAGCCGGCGCAAGCACGACCCAC
***MetY***	**F:** GGGGACAACTTTGTACAAAAAAGTTGGCATGGAGGCCCACCAGTTCATT **R:** GGGGACAACTTTGTACAAGAAAGTTGGTTAGCACTGTGCATAAAACTGTAG
***YPCP***	**F:** GGGGACAACTTTGTACAAAAAAGTTGGCCGCGCCGTCGTGACTTATGAG **R:** GGGGACAACTTTGTACAAGAAAGTTGGTTACAAAACATACTGTTCGGGACCGTTG
***MetYPCP (HEV-1)***	**F:** GGGGACAACTTTGTACAAAAAAGTTGGCATGGAGGCCCATCAGTTTATCAAG **R:** GGGGACAACTTTGTACAAGAAAGTTGGTTAAAGATTGTGGCGCTCCGGGC
***PCP (HEV-1)***	**F:** GGGGACAACTTTGTACAAAAAAGTTGGCCAGTGTAGGCGCTGGCTTTCG **R:** GGGGACAACTTTGTACAAGAAAGTTGGTTAAAGATTGTGGCGCTCCGGGC
***ISG56***	**F:** GGACAGGAAGCTGAAGGAG **R:** AGTGGGTGTTTCCTGCAA
***MDA5***	**F:** ACACGTTCTTTGCGATTTCC **R:** ACCAAATACAGGAGCCATGC
***OAS1***	**F:** CATCCGCCTAGTCAAGCACTG **R:** CACCACCCAAGTTTCCTGTAG
***GADPH***	**F:** GGTCGGAGGTCAACGGATTTG **R:** ACTCCACGACGTACTCAGCG

## Supplementary Materials

The following are available online at http://www.mdpi.com/1999-4915/10/12/726/s1, **Table S1**. Raw data from the luciferase reporter assays presented in Figure 1c. **Table S2**. Raw data from the luciferase reporter assays presented in Figure 5b. **Figure S1**. Expression of FLAG-tagged full-length and domains of ORF1 in co-transfected 293T cells. **Figure S2**. Relative expression of ISG56, MDA5 and OAS1 mRNA determined by RT-qPCR for each of the 3 independent experiments presented in Figure 2. **Figure S3**. Expression of ORF1, Y, X and Met of HEV ORF1 has no impact on STAT1 nuclear translocation upon IFN-β treatment. **Figure S4**. Effect of MetYPCP of HEV ORF1 on STAT1 translocation in response to IFN-II. **Figure S5**. Alignment of the amino acid sequence of MetYPCP from genotypes 1 and 3. **Figure S6**. Expression of FLAG-tagged MetYPCP and PCP from genotypes 1 and 3 in co-transfected 293T cells. **Figure S7**. Cell viability assay in cells expressing MetYPCP and PCP from HEV-1 and HEV-3. **Figure S8**. Effect of MetYPCP from HEV-1 on STAT1 translocation.
