# Supplementary material for: The Amino-Terminal Region of Hepatitis E Virus ORF1 Containing a Methyltransferase (Met) and a Papain-Like Cysteine Protease (PCP) Domain Counteracts Type I Interferon Response
Source: Viruses. 2018 Dec 18;10(12):726. doi: 10.3390/v10120726 (PMC6315852; doi:10.3390/v10120726)
Supplement: Supplementary file 1 [file viruses-10-00726-s001.pdf]

Supplementary Material

		Renilla (RLU)			Firefly (RLU)			Firefly/Renilla (F/R)			Mean (F/R)	%			Mean (%)	S.D. (%)
**Experiment 1**																
NT	EV	200300	201930	193190	300	340	450	0.0015	0.0017	0.0023	0.0018	2	3	4	**3**	0.7
	ORF1	-	520410	502360	-	2340	2280	-	0.0045	0.0045	0.0045	-	7	7	**7**	0.0
	MetYPCP	264980	282670	235400	290	320	190	0.0011	0.0011	0.0008	0.0010	2	2	1	**2**	0.3
	PCP	177860	200810	186360	220	220	350	0.0012	0.0011	0.0019	0.0014	2	2	3	**2**	0.7
	Met	136510	171450	148370	120	220	220	0.0009	0.0013	0.0015	0.0012	1	2	2	**2**	0.5
	MetY	241570	264610	261210	300	420	270	0.0012	0.0016	0.0010	0.0013	2	3	2	**2**	0.5
	YPCP	187780	170320	223080	370	260	450	0.0020	0.0015	0.0020	0.0018	3	2	3	**3**	0.4
	Y	324560	433740	414400	550	690	750	0.0017	0.0016	0.0018	0.0017	3	3	3	**3**	0.2
	X	233610	352240	365320	290	300	350	0.0012	0.0009	0.0010	0.0010	2	1	2	**2**	0.3
	MV-V	271880	268710	256650	200	300	220	0.0007	0.0011	0.0009	0.0009	1	2	1	**1**	0.3
IFN	EV	216550	222760	207050	14800	13890	11800	0.0683	0.0624	0.0570	0.0626	109	100	91	**100**	9.1
	ORF1	558780	579090	550160	24400	25720	22420	0.0437	0.0444	0.0408	0.0429	70	71	65	**69**	3.1
	MetYPCP	328990	291910	280530	6050	5840	6230	0.0184	0.0200	0.0222	0.0202	29	32	35	**32**	3.1
	PCP	214980	231230	233310	8470	9380	8770	0.0394	0.0406	0.0376	0.0392	63	65	60	**63**	2.4
	Met	209910	201380	191460	15920	14320	13570	0.0758	0.0711	0.0709	0.0726	121	114	113	**116**	4.5
	MetY	307740	297750	333400	14070	12090	14430	0.0457	0.0406	0.0433	0.0432	73	65	69	**69**	4.1
	YPCP	245040	252760	215480	15830	15870	14360	0.0646	0.0628	0.0666	0.0647	103	100	107	**103**	3.1
	Y	485760	520570	427280	25220	24900	19510	0.0519	0.0478	0.0457	0.0485	83	76	73	**77**	5.1
	X	442440	417760	424150	14950	17650	19960	0.0338	0.0422	0.0471	0.0410	54	68	75	**66**	10.7
	MV-V	314250	303040	312720	740	930	760	0.0024	0.0031	0.0024	0.0026	4	5	4	**4**	0.6
**Experiment 2**																
NT	EV	70170	66930	65930	130	80	150	0.00185	0.0012	0.00228	0.0018	9	6	11	**9**	2.7
	ORF1	149490	167270	167870	110	140	100	0.00074	0.00084	0.0006	0.0007	4	4	3	**4**	0.6
	MetYPCP	36800	29700	44740	60	60	60	0.00163	0.00202	0.00134	0.0017	8	10	7	**8**	1.7
	PCP	48340	43100	50030	40	90	20	0.00083	0.00209	0.0004	0.0011	4	10	2	**5**	4.3
	Met	29500	28030	30250	40	50	70	0.00136	0.00178	0.00231	0.0018	7	9	11	**9**	2.4
	MetY	46100	44970	50250	110	60	130	0.00239	0.00133	0.00259	0.0021	12	7	13	**10**	3.3
	YPCP	65090	81310	79390	60	110	140	0.00092	0.00135	0.00176	0.0013	5	7	9	**7**	2.1
	Y	55110	42850	46580	130	30	60	0.00236	0.0007	0.00129	0.0014	12	3	6	**7**	4.2
	X	112680	127890	119500	120	130	70	0.00106	0.00102	0.00059	0.0009	5	5	3	**4**	1.3
	MV-V	35540	40000	30880	90	100	20	0.00253	0.0025	0.00065	0.0019	13	12	3	**9**	5.3
IFN	EV	81100	90050	87250	1540	1610	2080	0.01899	0.01788	0.02384	0.02024	94	88	118	**100**	15.7
	ORF1	226430	222010	192670	2450	3660	2780	0.01082	0.01649	0.01443	0.01391	53	81	71	**69**	14.2
	MetYPCP	51430	60800	52650	790	800	740	0.01536	0.01316	0.01406	0.01419	76	65	69	**70**	5.5
	PCP	46860	43900	46970	970	1190	1270	0.0207	0.02711	0.02704	0.02495	102	134	134	**123**	18.2
	Met	37880	49790	45390	1180	1190	1190	0.03115	0.0239	0.02622	0.02709	154	118	130	**134**	18.3
	MetY	54120	57500	65730	1850	2100	2540	0.03418	0.03652	0.03864	0.03645	169	180	191	**180**	11.0
	YPCP	92860	93870	77410	2280	2420	1860	0.02455	0.02578	0.02403	0.02479	121	127	119	**122**	4.4
	Y	84040	81300	77870	1870	1660	1840	0.02225	0.02042	0.02363	0.02210	110	101	117	**109**	8.0
	X	183290	152810	116600	3450	2990	2390	0.01882	0.01957	0.0205	0.01963	93	97	101	**97**	4.1
	MV-V	49480	75870	57270	100	130	60	0.00202	0.00171	0.00105	0.00159	10	8	5	**8**	2.5
**Experiment 3**																
NT	EV	202890	217220	173590	250	450	250	0.00123	0.00207	0.00144	0.00158	4	6	4	**5**	1.4
	ORF1	329300	344920	331430	1430	1500	1200	0.00434	0.00435	0.00362	0.00410	13	13	11	**13**	1.3
	MetYPCP	306760	303370	280160	130	170	210	0.00042	0.00056	0.00075	0.00058	1	2	2	**2**	0.5
	PCP	121330	145490	172800	130	170	290	0.00107	0.00117	0.00168	0.00131	3	4	5	**4**	1.0
	Met	173750	179090	158860	180	220	210	0.00104	0.00123	0.00132	0.00120	3	4	4	**4**	0.5
	MetY	284940	361920	361530	350	440	460	0.00123	0.00122	0.00127	0.00124	4	4	4	**4**	0.1
	YPCP	278550	268710	260290	370	220	330	0.00133	0.00082	0.00127	0.00114	4	3	4	**4**	0.9
	Y	192870	293460	313440	230	330	440	0.00119	0.00112	0.00140	0.00124	4	3	4	**4**	0.5
	X	39720	500870	381910	20	340	230	0.00050	0.00068	0.00060	0.00059	2	2	2	**2**	0.3
	MV-V	392070	472870	431490	140	230	230	0.00036	0.00049	0.00053	0.00046	1	2	2	**1**	0.3
IFN	EV	186360	188650	207150	5390	6380	7110	0.02892	0.03382	0.03432	0.03235	89	105	106	**100**	9.2
	ORF1	331090	407430	411700	11090	13640	15280	0.03350	0.03348	0.03711	0.03470	104	103	115	**107**	6.5
	MetYPCP	302210	317880	370720	1800	1470	1820	0.00596	0.00462	0.00491	0.00516	18	14	15	**16**	2.2
	PCP	151110	157330	167870	4460	4000	4730	0.02951	0.02542	0.02818	0.02771	91	79	87	**86**	6.4
	Met	199150	181240	173830	8380	9060	8410	0.04208	0.04999	0.04838	0.04682	130	155	150	**145**	12.9
	MetY	404720	417510	428310	7850	7970	7740	0.01940	0.01909	0.01807	0.01885	60	59	56	**58**	2.1
	YPCP	307040	306240	338200	6900	7350	7990	0.02247	0.02400	0.02363	0.02337	69	74	73	**72**	2.5
	Y	341030	353540	344960	10160	12200	10530	0.02979	0.03451	0.03053	0.03161	92	107	94	**98**	7.8
	X	606560	569470	711570	10210	8720	11720	0.01683	0.01531	0.01647	0.01621	52	47	51	**50**	2.5
	MV-V	540820	599960	571560	510	600	460	0.00094	0.00100	0.00080	0.00092	3	3	2	**3**	0.3
**Experiment 4**																
NT	EV	137220	149460	135880	170	220	180	0.00124	0.00147	0.00132	0.00135	2	2	2	**2**	0.2
	ORF1	189840	186430	187930	2180	4070	2210	0.01148	0.02183	0.01176	0.01502	19	37	20	**25**	10.0
	MetYPCP	127670	126210	147810	130	260	270	0.00102	0.00206	0.00183	0.00163	2	3	3	**3**	0.9
	PCP	91050	101560	84190	150	170	130	0.00165	0.00167	0.00154	0.00162	3	3	3	**3**	0.1
	Met	88370	92430	91270	210	170	190	0.00238	0.00184	0.00208	0.0021	4	3	4	**4**	0.5
	MetY	117910	97530	121100	320	190	240	0.00271	0.00195	0.00198	0.00221	5	3	3	**4**	0.7
	YPCP	108680	108150	94910	160	210	290	0.00147	0.00194	0.00306	0.00216	2	3	5	**4**	1.4
	Y	183030	136090	171300	410	570	530	0.00224	0.00419	0.00309	0.00317	4	7	5	**5**	1.7
	X	27010	115520	133490	40	150	360	0.00148	0.0013	0.0027	0.00183	3	2	5	**3**	1.3
	MV-V	134080	124570	127210	180	160	200	0.00134	0.00128	0.00157	0.0014	2	2	3	**2**	0.3
IFN	EV	170650	169920	149520	9170	9290	10250	0.05374	0.05467	0.06855	0.05899	91	93	116	**100**	14.1
	ORF1	229380	246710	192860	19060	17490	15540	0.08309	0.07089	0.08058	0.07819	141	120	137	**133**	10.9
	MetYPCP	137030	138520	121100	5190	5020	5660	0.03787	0.03624	0.04674	0.04028	64	61	79	**68**	9.6
	PCP	111450	105190	112120	4750	3720	4160	0.04262	0.03536	0.0371	0.03836	72	60	63	**65**	6.4
	Met	112430	111490	103620	9780	9090	8710	0.08699	0.08153	0.08406	0.08419	147	138	143	**143**	4.6
	MetY	117760	127700	113820	15340	12990	11690	0.13026	0.10172	0.10271	0.11156	221	172	174	**189**	27.5
	YPCP	115350	110510	100400	11300	13010	9790	0.09796	0.11773	0.09751	0.1044	166	200	165	**177**	19.6
	Y	173340	176590	171730	11790	12470	10880	0.06802	0.07062	0.06336	0.06733	115	120	107	**114**	6.2
	X	139850	132560	134610	12310	11320	12720	0.08802	0.0854	0.0945	0.0893	149	145	160	**151**	7.9
	MV-V	132770	136310	128020	460	640	520	0.00346	0.0047	0.00406	0.00407	6	8	7	**7**	1.0

Table S1. Raw data from the luciferase reporter assays presented in Figure 1c. Mean ratios between firefly and *Renilla* luciferase activities were calculated (F/R) and are presented as percentages (%) of the treated EV control in 4 independent experiments. Mean values and standard deviation (S.D.) are presented for not-treated (NT) and IFN-β-treated (IFN) conditions.

		Renilla (RLU)			Firefly (RLU)			Firefly/Renilla (F/R)			Mean (F/R)	%			Mean (%)	S.D. (%)
Experiment 1																
NT	EV	200300	201930	193190	300	340	450	0.0015	0.0017	0.0023	0.0018	2	3	4	**3**	0.7
	MetYPCP-G3	264980	282670	235400	290	320	190	0.0011	0.0011	0.0008	0.0010	2	2	1	**2**	0.3
	PCP-G3	177860	200810	186360	220	220	350	0.0012	0.0011	0.0019	0.0014	2	2	3	**2**	0.7
	MetYPCP-G1	202530	207220	222490	360	310	300	0.0018	0.0015	0.0013	0.0015	3	2	2	**2**	0.3
	PCP-G1	148850	212410	210900	260	260	360	0.0017	0.0012	0.0017	0.0016	3	2	3	**2**	0.5
	MV-V	271880	268710	256650	200	300	220	0.0007	0.0011	0.0009	0.0009	1	2	1	**1**	0.3
IFN	EV	216550	222760	207050	14800	13890	11800	0.0683	0.0624	0.0570	0.0626	109	100	91	**100**	9.1
	MetYPCP-G3	328990	291910	280530	6050	5840	6230	0.0184	0.0200	0.0222	0.0202	29	32	35	**32**	3.1
	PCP-G3	214980	231230	233310	8470	9380	8770	0.0394	0.0406	0.0376	0.0392	63	65	60	**63**	2.4
	MetYPCP-G1	268250	245390	287010	10810	8390	10550	0.0403	0.0342	0.0368	0.0371	64	55	59	**59**	4.9
	PCP-G1	245420	228780	221960	16700	18660	14520	0.0680	0.0816	0.0654	0.0717	109	130	105	**115**	13.8
	MV-V	314250	303040	312720	740	930	760	0.0024	0.0031	0.0024	0.0026	4	5	4	**4**	0.6
Experiment 2																
NT	EV	70170	66930	65930	130	80	150	0.00185	0.0012	0.00228	0.0018	9	6	11	**9**	2.7
	MetYPCP-G3	36800	29700	44740	60	60	60	0.00163	0.00202	0.00134	0.0017	8	10	7	**8**	1.7
	PCP-G3	48340	43100	50030	40	90	20	0.00083	0.00209	0.0004	0.0011	4	10	2	**5**	4.3
	MetYPCP-G1	45740	37020	43580	60	90	100	0.00131	0.00243	0.00229	0.0020	6	12	11	**10**	3.0
	PCP-G1	60430	54670	61390	80	70	70	0.00132	0.00128	0.00114	0.0012	7	6	6	**6**	0.5
	MV-V	35540	40000	30880	90	100	20	0.00253	0.0025	0.00065	0.0019	13	12	3	**9**	5.3
IFN	EV	81100	90050	87250	1540	1610	2080	0.01899	0.01788	0.02384	0.02024	94	88	118	**100**	15.7
	MetYPCP-G3	51430	60800	52650	790	800	740	0.01536	0.01316	0.01406	0.01419	76	65	69	**70**	5.5
	PCP-G3	46860	43900	46970	970	1190	1270	0.0207	0.02711	0.02704	0.02495	102	134	134	**123**	18.2
	MetYPCP-G1	54080	60920	56870	1440	1730	1420	0.02663	0.0284	0.02497	0.02666	132	140	123	**132**	8.5
	PCP-G1	59910	66630	63450	1380	1420	1780	0.02303	0.02131	0.02805	0.02413	114	105	139	**119**	17.3
	MV-V	49480	75870	57270	100	130	60	0.00202	0.00171	0.00105	0.00159	10	8	5	**8**	2.5
Experiment 3																
NT	EV	202890	217220	173590	250	450	250	0.00123	0.00207	0.00144	0.00158	4	6	4	**5**	1.4
	MetYPCP-G3	306760	303370	280160	130	170	210	0.00042	0.00056	0.00075	0.00058	1	2	2	**2**	0.5
	PCP-G3	121330	145490	172800	130	170	290	0.00107	0.00117	0.00168	0.00131	3	4	5	**4**	1.0
	MetYPCP-G1	247460	244000	277580	160	130	180	0.00065	0.00053	0.00065	0.00061	2	2	2	**2**	0.2
	PCP-G1	355340	499600	352410	260	360	240	0.00073	0.00072	0.00068	0.00071	2	2	2	**2**	0.1
	MV-V	392070	472870	431490	140	230	230	0.00036	0.00049	0.00053	0.00046	1	2	2	**1**	0.3
IFN	EV	186360	188650	207150	5390	6380	7110	0.02892	0.03382	0.03432	0.03235	89	105	106	**100**	9.2
	MetYPCP-G3	302210	317880	370720	1800	1470	1820	0.00596	0.00462	0.00491	0.00516	18	14	15	**16**	2.2
	PCP-G3	151110	157330	167870	4460	4000	4730	0.02951	0.02542	0.02818	0.02771	91	79	87	**86**	6.4
	MetYPCP-G1	335600	282950	299520	3110	3240	3230	0.00927	0.01145	0.01078	0.01050	29	35	33	**32**	3.5
	PCP-G1	412860	393580	372060	6850	5680	5980	0.01659	0.01443	0.01607	0.01570	51	45	50	**49**	3.5
	MV-V	540820	599960	571560	510	600	460	0.00094	0.00100	0.00080	0.00092	3	3	2	**3**	0.3
Experiment 4																
NT	EV	137220	149460	135880	170	220	180	0.00124	0.00147	0.00132	0.0013452	2	2	2	**2**	0.2
	MetYPCP-G3	127670	126210	147810	130	260	270	0.00102	0.00206	0.00183	0.001635	2	3	3	**3**	0.9
	PCP-G3	91050	101560	84190	150	170	130	0.00165	0.00167	0.00154	0.0016218	3	3	3	**3**	0.1
	MetYPCP-G1	298560	298620	303850	310	240	210	0.00104	0.0008	0.00069	0.0008444	2	1	1	**1**	0.3
	PCP-G1	121710	112650	111290	300	310	440	0.00246	0.00275	0.00395	0.0030568	4	5	7	**5**	1.3
	MV-V	134080	124570	127210	180	160	200	0.00134	0.00128	0.00157	0.0013997	2	2	3	**2**	0.3
IFN	EV	170650	169920	149520	9170	9290	10250	0.05374	0.05467	0.06855	0.0589871	91	93	116	**100**	14.1
	MetYPCP-G3	137030	138520	121100	5190	5020	5660	0.03787	0.03624	0.04674	0.0402845	64	61	79	**68**	9.6
	PCP-G3	111450	105190	112120	4750	3720	4160	0.04262	0.03536	0.0371	0.0383626	72	60	63	**65**	6.4
	MetYPCP-G1	312570	304910	274430	10090	8540	9880	0.03228	0.02801	0.036	0.032097	55	47	61	**54**	6.8
	PCP-G1	125860	122480	113670	11860	12790	9390	0.09423	0.10443	0.08261	0.0937548	160	177	140	**159**	18.5
	MV-V	132770	136310	128020	460	640	520	0.00346	0.0047	0.00406	0.0040739	6	8	7	**7**	1.0
Experiment 5																
NT	EV	34730	61960	53620	60	250	140	0.00173	0.00403	0.00261	0.0027911	2	5	3	**3**	1.4
	MetYPCP-G3	106760	106090	114750	180	200	240	0.00169	0.00189	0.00209	0.0018876	2	2	3	**2**	0.3
	PCP-G3	59960	57460	69510	140	100	160	0.00233	0.00174	0.0023	0.0021257	3	2	3	**3**	0.4
	MetYPCP-G1	55420	54140	62980	200	250	280	0.00361	0.00462	0.00445	0.0042241	4	6	5	**5**	0.7
	PCP-G1	46030	54840	50000	190	250	150	0.00413	0.00456	0.003	0.0038955	5	6	4	**5**	1.0
	MV-V	63230	55010	51370	120	100	100	0.0019	0.00182	0.00195	0.0018874	2	2	2	**2**	0.1
IFN	EV	70050	69220	61310	5650	5120	5390	0.08066	0.07397	0.08791	0.0808459	100	91	109	**100**	8.6
	MetYPCP-G3	130380	125220	99210	4130	3710	3680	0.03168	0.02963	0.03709	0.0327992	39	37	46	**41**	4.8
	PCP-G3	84470	70890	55510	4420	4310	3980	0.05233	0.0608	0.0717	0.0616078	65	75	89	**76**	12.0
	MetYPCP-G1	68850	72360	60140	5070	5070	4430	0.07364	0.07007	0.07366	0.0724554	91	87	91	**90**	2.6
	PCP-G1	66090	56780	53050	5350	3860	4170	0.08095	0.06798	0.07861	0.0758457	100	84	97	**94**	8.5
	MV-V	58160	49940	53740	750	550	670	0.0129	0.01101	0.01247	0.0121254	16	14	15	**15**	1.2

Table S2. Raw data from the luciferase reporter assays presented in Figure 6b. Mean ratios between firefly and *Renilla* luciferase activities were calculated (F/R) and are presented as percentages (%) of the treated EV control in 5 independent experiments. Mean values and standard deviation (S.D.) are presented for not-treated (NT) and IFN-β-treated (IFN) conditions.

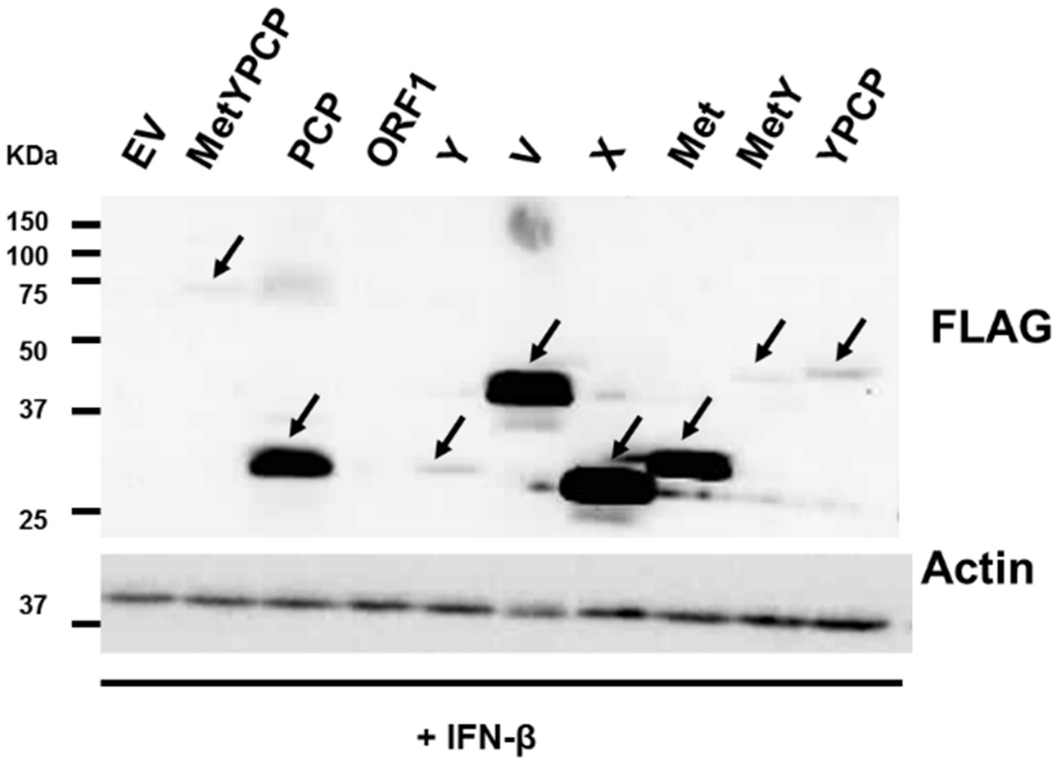

Figure S1. Expression of FLAG-tagged full-length and domains of ORF1 in co-transfected 293T cells. Cell lysates generated for the luciferase reporter assay (Figure 1c) were analysed by immunoblotting using an anti-FLAG antibody. Bands corresponding to the different FLAG-tagged proteins are shown by arrows. Note that ORF1 was not detected. Actin served as loading control.

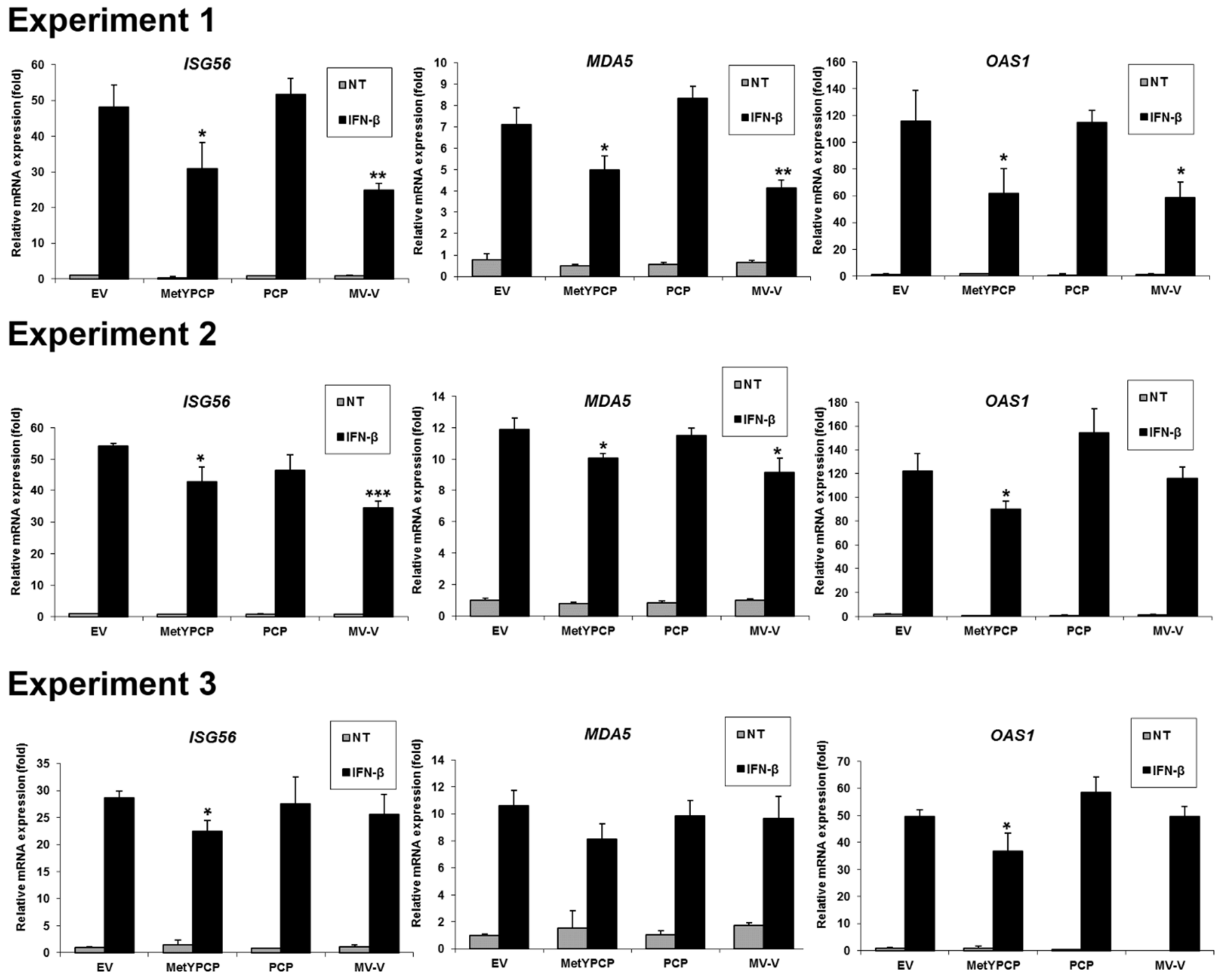

Figure S2. Relative expression of ISG56, MDA5 and OAS1 mRNA determined by RT-qPCR for each of the 3 independent experiments presented in Figure 2. Data are presented as fold induction (+ standard deviations) relative to the non-stimulated EV control. *, P < 0.05; **, P < 0.005; ***, P < 0.0005 compared to EV control for treated samples (unpaired t tests).

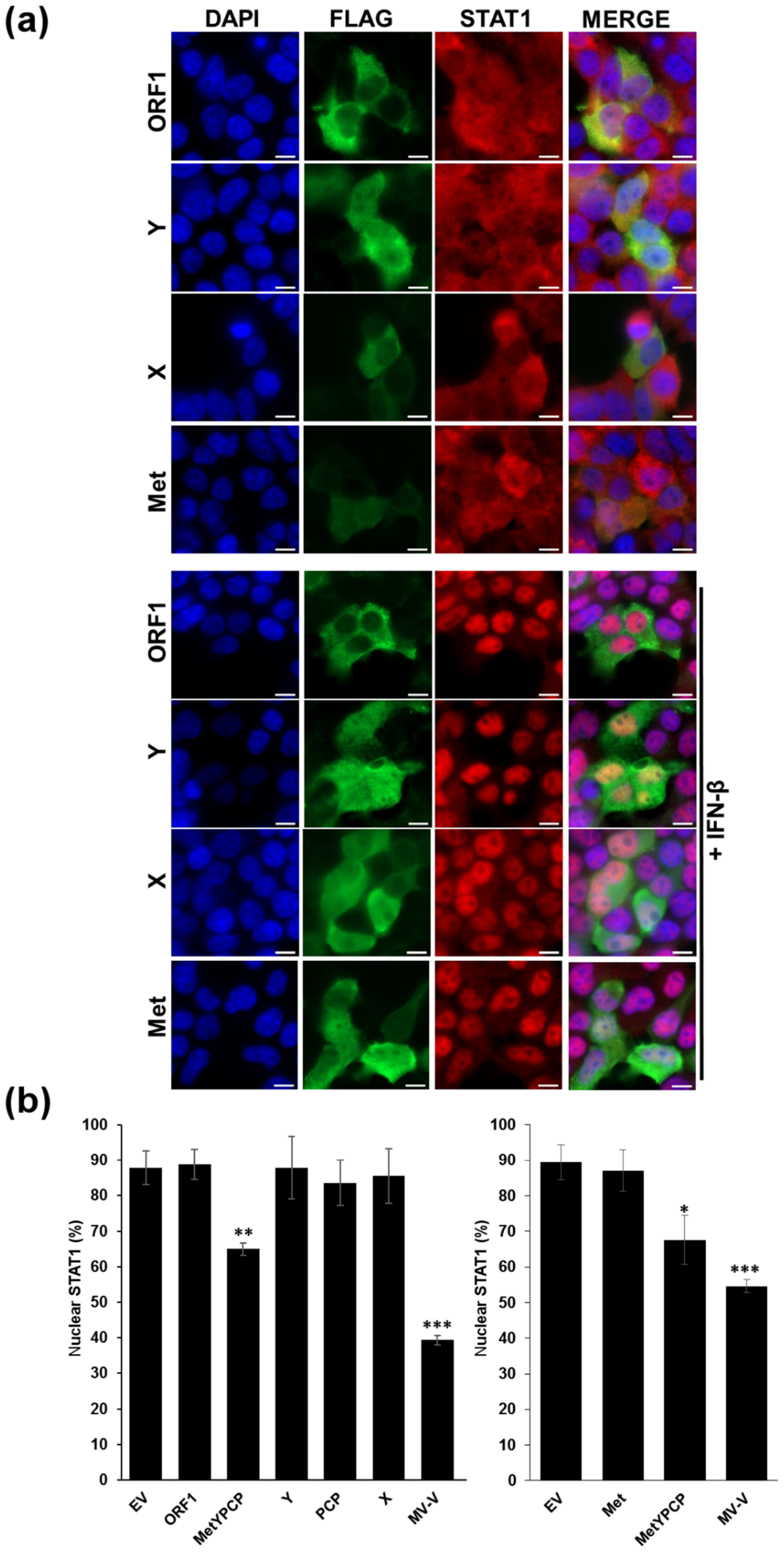
(a)
DAPI
FLAG
STAT1
MERGE
ORF1
Y
X
Met
ORF1
Y
X
Met
+ IFN-β
(b)
Nuclear STAT1 (%)
100
90
80
70
60
50
40
30
20
10
0
**
***
EV
ORF1
MetYPCP
Y
PCP
X
MV-V
Nuclear STAT1 (%)
100
90
80
70
60
50
40
30
20
10
0
*
***
EV
Met
MetYPCP
MV-V

Figure S3. Expression of ORF1, Y, X and Met of HEV ORF1 has no impact on STAT1 nuclear translocation upon IFN-β treatment. (**a**) 293T cells were transfected with a pCINeo-3xFLAG empty vector (EV) or a plasmid coding for ORF1, Y, X or Met fused to a FLAG tag. Twenty four h post-transfection, cells were left untreated or stimulated for 30 min with 1000 IU/ml of IFN-β. Cells were then washed, fixed and stained with primary antibodies raised against STAT1 and FLAG, followed by fluorescent dye-conjugated secondary antibodies. Intracellular localization of DAPI-stained nuclei (blue), FLAG (green) and STAT1 (red) was visualized by microscopy (magnification, x630). Scale bars, 10 µm. (**b**) STAT1 localization was visualized after immunostaining as described in (a) in 293T cells transfected with a pCINeo-3xFLAG empty vector (EV) or a plasmid coding for ORF1, MetYPCP, PCP, X, Y, Met or MV-V fused to a FLAG tag. For each condition, STAT1 localization was determined in 48 to 181 cells expressing the corresponding FLAG-tagged protein (except for the EV control for which 356 to 384 cells were randomly assessed). The mean percentage (± standard deviation) of cells showing a predominant nuclear localization of STAT1 from 3 independent experiments is shown. *$p<0.05$; **$p<0.005$; ***$p<0.0005$ compared to EV control for treated samples (unpaired t tests). **(a-b)** Data presented for the Met domain are from a different set of experiments than the one presented for ORF1, Y and X.

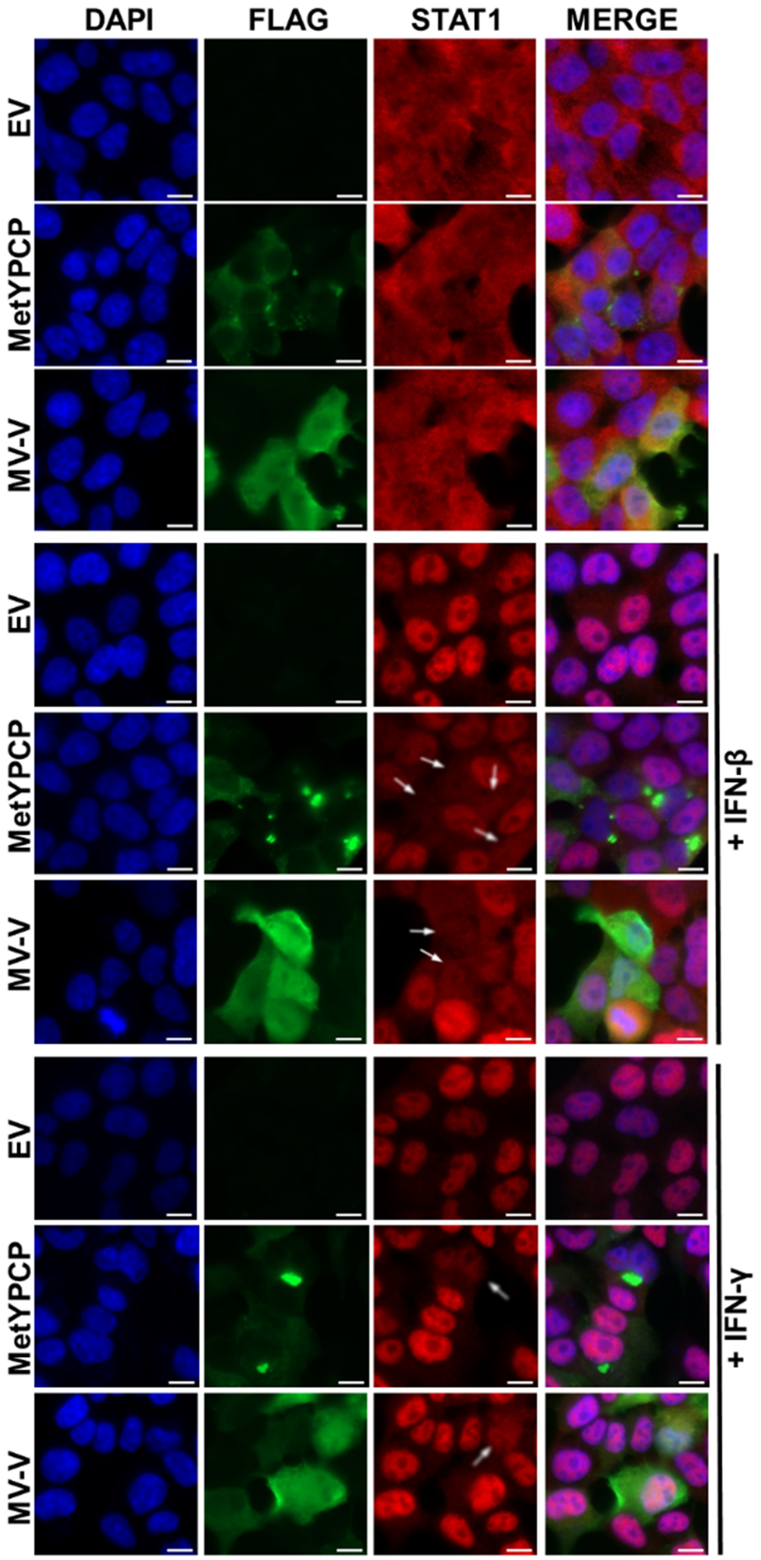

Figure S4. Effect of MetYPCP of HEV ORF1 on STAT1 translocation in response to IFN-II. 293T cells were transfected with a pCINeo-3×FLAG empty vector (EV) or a plasmid coding for MetYPCP or MV-V fused to a 3xFLAG tag. Twenty four h post-transfection, cells were stimulated for 30 min with 1000 IU/ml of IFN-β or 250 ng/ml of IFN-γ. Cells were then washed, fixed and stained with primary antibodies raised against STAT1 and FLAG, followed by fluorescent dye-conjugated secondary antibodies. Cells showing diffuse cytoplasmic/nuclear localisation of STAT1 upon IFN-β treatment are indicated by arrows. Scale bars, 10 μm.

```
MetYPCP-G1   1 MEAHQFIKAPGITTAIEQAALAAANSALANAVVVRPFLSHQQIEILINLM   50
               |||||||||||||||||||||||||||||||||||||||..|.:||||||
MetYPCP-G3   1 MEAHQFIKAPGITTAIEQAALAAANSALANAVVVRPFLSRVQTDILINLM   50

MetYPCP-G1  51 QPRQLVFRPEVFWNHPIQRVIHNELELYCRARSGRCLEIGAHPRSINDNP  100
               |||||||||||.||||||||||||||.|||||:|||||:|||||||||||
MetYPCP-G3  51 QPRQLVFRPEVLWNHPIQRVIHNELEQYCRARAGRCLEVGAHPRSINDNP  100

MetYPCP-G1 101 NVVHRCFLRPVGRDVQRWYTAPTRGPAANCRRSALRGLPAADRTYCFDGF  150
               ||:||||||||||||||||:|||||||||||||||||||..|||||||||
MetYPCP-G3 101 NVLHRCFLRPVGRDVQRWYSAPTRGPAANCRRSALRGLPPVDRTYCFDGF  150

MetYPCP-G1 151 SGCNFPAETGIALYSLHDMSPSDVAEAMFRHGMTRLYAALHLPPEVLLPP  200
               |.|.|.||||||||||||:.||||||||.|||||||||||||||||||||
MetYPCP-G3 151 SRCAFAAETGIALYSLHDLWPSDVAEAMARHGMTRLYAALHLPPEVLLPP  200

MetYPCP-G1 201 GTYRTASYLLIHDGRRVVVTYEGDTSAGYNHDVSNLRSWIRTTKVTGDHP  250
               |||.|.||||||||.|.|||||||||||||||||.||:||||||:.||||
MetYPCP-G3 201 GTYHTTSYLLIHDGNRAVVTYEGDTSAGYNHDVSILRAWIRTTKIVGDHP  250

MetYPCP-G1 251 LVIERVRAIGCHFVLLLTAAPEPSPMPYVPYPRSTEVYVRSIFGPGGTPS  300
               |||||||||||||||||||||||||||||||||||||||||||||||:||
MetYPCP-G3 251 LVIERVRAIGCHFVLLLTAAPEPSPMPYVPYPRSTEVYVRSIFGPGGSPS  300

MetYPCP-G1 301 LFPTSCSTKSTFHAVPTHIWDRLMLFGATLDDQAFCCSRLMTYLRGISYK  350
               |||::|||||||||||.|||||||||||||||||||||||||||||||||
MetYPCP-G3 301 LFPSACSTKSTFHAVPVHIWDRLMLFGATLDDQAFCCSRLMTYLRGISYK  350

MetYPCP-G1 351 VTVGTLVANEGWNASEDALTAVITAAYLTICHQRYLRTQAISKGMRRLER  400
               ||||.||||||||||||||||||||||||||||||||||||||||||||.
MetYPCP-G3 351 VTVGALVANEGWNASEDALTAVITAAYLTICHQRYLRTQAISKGMRRLEV  400

MetYPCP-G1 401 EHAQKFITRLYSWLFEKSGRDYIPGRQLEFYAQCRRWLSAGFHLDPRVLV  450
               ||||||||||||||||||||||||||||:|||||||||||||||||||||
MetYPCP-G3 401 EHAQKFITRLYSWLFEKSGRDYIPGRQLQFYAQCRRWLSAGFHLDPRVLV  450

MetYPCP-G1 451 FDESAPCHCRTAIRKAFSKFCCFMKWLGQECTCFLQPAEGVVGDQGHDNE  500
               |||:.||.||:.::|...|||||||||||||||||:||||:|||.|||||
MetYPCP-G3 451 FDEAVPCRCRSFLKKVAGKFCCFMKWLGQECTCFLEPAEGLVGDCGHDNE  500

MetYPCP-G1 501 AYEGSDVDPAESAISDISGSYVVPGTALQPLYQALDLPAEIVARAGRLTA  550
               |||||:|||||.|..|:||:|.|.|..|:.||:||::|.:|.|||.||||
MetYPCP-G3 501 AYEGSEVDPAEPAHLDVSGTYAVHGRQLEALYRALNIPDDIAARASRLTA  550

MetYPCP-G1 551 TVKVSQVDGRIDCETLLGNKTFRTSFVDGAVLETNGPERHNL  592
               ||:::...||::|.|:|||||||||..|||.||.||||::.|
MetYPCP-G3 551 TVELAASPGRLECRTVLGNKTFRTSITDGAHLEANGPEQYVL  592
```

Figure S5. Alignment of the amino acid sequence of MetYPCP from genotypes 1 and 3. The amino acid sequence of MetYPCP from genotype 1 (MetYPCP-G1) is 86% identical to the one of MetYPCP from genotype 3 (MetYPCP-G3). Pairwise Sequence Alignment (PROTEIN) EMBOSS Needle was used (https://www.ebi.ac.uk/Tools/psa/emboss_needle/). Different amino acids are highlighted in red. : indicates similar amino acids.

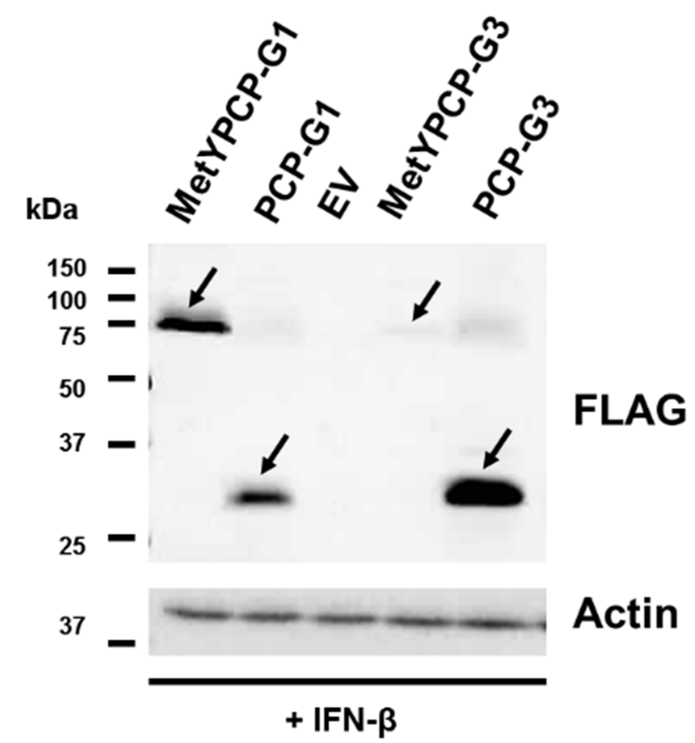

Figure S6. Expression of FLAG-tagged MetYPCP and PCP from genotypes 1 and 3 in co-transfected 293T cells. Cell lysates generated for the luciferase reporter assay (Figure 6b) were analysed by immunoblotting using an anti-FLAG antibody. Bands corresponding to the different FLAG-tagged proteins are shown by arrows. Actin served as loading control.

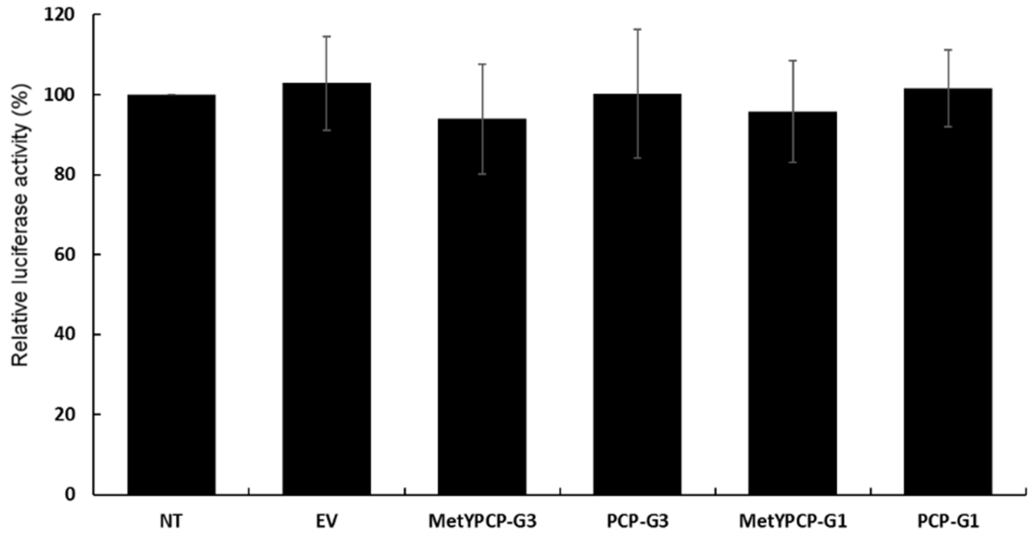

Figure S7. Cell viability assay in cells expressing MetYPCP and PCP from HEV-1 and HEV-3. 293T cells were transfected or not (NT) with a pCINeo-3xFLAG empty vector (EV) or a plasmid coding for MetYPCP and PCP from genotype 1 and 3 fused to a 3xFLAG tag. Forty h after transfection, cells were lysed and cell viability determined using a luminescent-based assay. Luciferase activities (± standard deviations) are expressed as percentage relative to non-transfected cells. No significant difference was found between the cells transfected with the pCINeo-3xFLAG empty vector and the one transfected with the plasmids coding for MetYPCP or PCP from HEV-1 and HEV-3 (unpaired t tests). Results represent the mean of 3 independent experiments performed in triplicate.

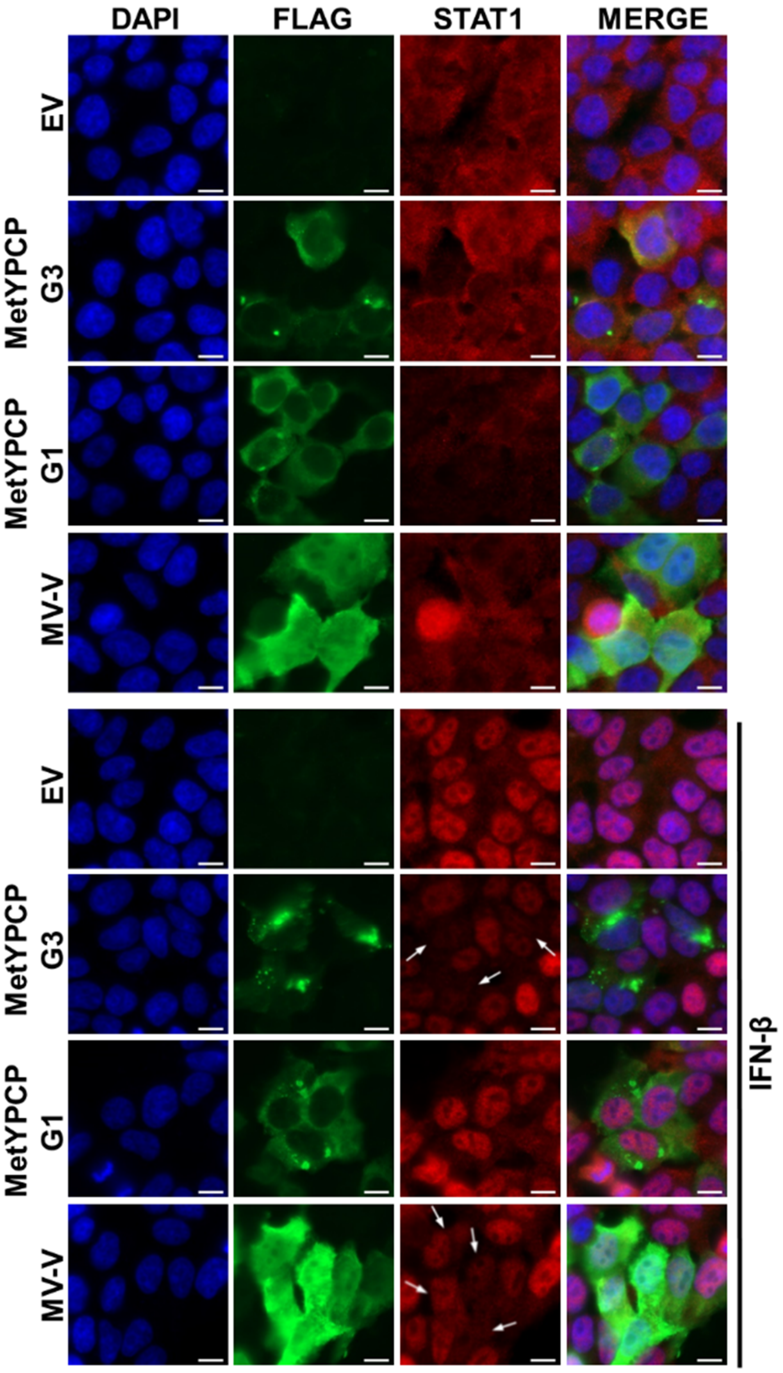

Figure S8. Effect of MetYPCP from HEV-1 on STAT1 translocation. 293T cells were transfected with a pCINeo-3xFLAG empty vector (EV) or a plasmid coding for MetYPCP from HEV-1 or HEV-3 or MV-V fused to a 3xFLAG tag. Twenty-four h post-transfection, cells were stimulated for 30 min with 1000 IU/ml of IFN-β. Cells were then washed, fixed and stained with primary antibodies raised against STAT1 and FLAG, followed by fluorescent dye-conjugated secondary antibodies. Cells showing diffuse STAT1 cytoplasmic/nuclear localisation upon IFN-β treatment are indicated by arrows. Scale bars, 10 μm.
